# Risk of hospitalization from drug-drug interactions in the Elderly: real-world evidence in a large administrative database

**DOI:** 10.18632/aging.104018

**Published:** 2020-10-05

**Authors:** Floor Swart, Giampaolo Bianchi, Jacopo Lenzi, Marica Iommi, Lorenzo Maestri, Emanuel Raschi, Marco Zoli, Fabrizio De Ponti, Elisabetta Poluzzi

**Affiliations:** 1 School of Medicine, Vrije University of Amsterdam, Amsterdam, The Netherlands; 2 Department of Medical and Surgical Sciences, University of Bologna, Bologna, Italy; 3 Centre of Studies and Research on the Elderly, University of Bologna, Bologna, Italy; 4 Department of Biomedical and Neuromotor Sciences, University of Bologna, Bologna, Italy

**Keywords:** drug-drug interactions, adverse drug reactions, elderly, case-control study, real-world evidence

## Abstract

The aim of this study was to assess the risk of hospitalization associated with the concomitant prescription of 10 highly prevalent drug-drug interactions (DDIs) among all individuals aged ≥65 residing in Bologna’s area, Italy. We used incidence density sampling, and the effect of current (last month) and past (≥30 days before) exposure to DDI was investigated through conditional multivariable logistic regression analysis.

Two DDIs were associated with increased hospitalization due to DDI related conditions: ACE-inhibitors/ diuretics plus glucocorticoids (current DDI: OR 2.36, 95% CI 1.94-2.87; past DDI: OR 1.36, 95% CI 1.12-1.65) and antidiabetic therapy plus current use of fluoroquinolones (OR 4.43, 95% CI 1.61-11.2). Non-Steroidal Anti-inflammatory Drugs (NSAIDs) increased the risk of re-bleeding in patients taking Selective Serotonin Reuptake Inhibitors (OR 5.56, 95% CI 1.24-24.9), while no significant effect was found in those without a history of bleeding episodes. Concomitant prescription of NSAIDs and ACE-inhibitors/diuretics in patients with a history of high-risk conditions was infrequent.

Within the pattern of drug prescriptions in the older population of Bologna’s area, we distinguished DDIs with actual clinical consequences from others that might be considered generally safe. Observed prescribing habits of clinicians reflect awareness of potential interactions in patients at risk.

## INTRODUCTION

Elderly patients are particularly susceptible to adverse drug reactions (ADRs) because of changes in pharmacokinetics and/or pharmacodynamics occurring with aging [1]. The higher prevalence of chronic diseases in these patients leads to increased drug consumption and greater number of adverse events, including those caused by drug-drug-interactions (DDIs).

In order to minimize the number of DDIs in this population, lists such as the American Geriatrics Society Beers Criteria® (AGS Beers Criteria®) have been developed to assist clinicians in prescribing the most appropriate drugs, while recent literature has focused on (promising) tools to identify, describe and limit potential DDIs through electronic decision support systems or pharmacist led-interventions [2–4].

Nevertheless, the prevalence of DDIs is high and has been the cause of the increasing incidence of hospitalization of older patients over the past decade [1, 5–9]. Notably in view of their vulnerability due to comorbidities such as cardiovascular diseases, it is often difficult to clearly attribute hospital admission to the concomitant use of interacting drugs. Therefore, older patients are often excluded from studies, although they are presumably most susceptible to ADRs.

In order to properly assess the benefit-risk ratio when prescribing drugs, clinicians need evidence of the actual risk of DDIs in real-world population, including patients with increased vulnerability.

The aim of this study based on real-world data was to assess the risk of hospitalization associated with the concomitant prescription of 10 highly prevalent DDIs in Bologna area for all individuals aged ≥65, thereby including also patients with increased vulnerability.

## RESULTS

The number of subjects included in the study changed greatly according to the chronic drugs of interest: more specifically, population sizes ranged from 146,418 for antihypertensives to 7249 for NOACs. The diagrams depicting selection of the study populations for each interaction analysis are reported in Supplementary Figures 1–10. As shown in Table 1, the overall incidence rates of hospitalization observed in the cohorts eligible for matching ranged from 0.6 (analysis #4 [SSRIs plus NSAIDs]; analysis #10 [SSRIs plus ASA] to 8.4 per 1000 person-months (analysis #3, diuretics plus NSAIDs).

**Table 1:** Overall incidence rates of hospitalization due to conditions potentially induced by DDI.

**#**	**Interaction analysis**	Hospital admissions*	**Person-months of follow-up**	**Incidence rate (95% CI) per 1000 person-months**
1	ACEIs/ARBs plus NSAIDs	1935	652,862.8	3.0 (2.8-3.1)
2	ACEIs/ARBs or Diuretics plus glucocorticoids	2300	713,899.0	3.2 (3.1-3.4)
3	Diuretics plus NSAIDs	1523	182,199.3	8.4 (7.9-8.8)
4	SSRIs plus NSAIDs	81	128,789.2	0.6 (0.5-0.8)
5	Vitamin K antagonists plus NSAIDs	98	76,188.3	1.3 (1.0-1.6)
6	NOACs plus NSAIDs	32	42,339.5	0.8 (0.5-1.1)
7	Vitamin K antagonists plus antibiotics/antimycotics	98	76,188.3	1.3 (1.0-1.6)
8	Antihypertensive plus α-blockers	1617	850,808.1	1.9 (1.8-2.0)
9	Antidiabetics plus fluoroquinolones	135	173,630.1	0.8 (0.7-0.9)
10	SSRIs plus ASA	81	128,789.2	0.6 (0.5-0.8)

* DDI-related conditions requiring hospital admissions are listed in Supplementary Table 1.

*CI*, confidence interval.

Table 2 shows the demographic and clinical characteristics of the cases (i.e., patients hospitalized during follow-up) and matched controls. The mean age of cases was 82.2±7.6 years, while mean time-to-event was 2.7±1.8 months. Most cases were found in analysis #2 (ACEIs/ARBs or diuretics plus glucocorticoids, *n*=1993), #1 (ACEIs/ARBs plus NSAIDs, *n*=1687), #8 (antihypertensives plus α-blockers, *n*=1407) and #3 (diuretics plus NSAIDs, *n*=1101). In the other interaction analyses the number of cases was much lower, ranging from 28 (analysis #6, NOACs plus NSAIDs) to 110 (analysis #9, antidiabetics plus fluoroquinolones). The distribution of sex, age, follow-up duration and history of high-risk comorbidities was virtual identical in cases and controls, thereby confirming proper matching on these variables. Compared to the controls, cases made more extensive use of antidiabetics and interfering medications, and had higher Elixhauser comorbidity scores; except for analyses #5 to #7 (vitamin K antagonists and NOACs), cases took >4 concurrent drugs more frequently. In analysis #3 (diuretics plus NSAIDs) there was the highest number of interfering drug users (>80%).

**Table 2:** Characteristics of patients hospitalized during follow-up (cases) and matched controls, by interaction analysis. Values are counts (percentages) or mean [standard deviation].

**# Interaction analysis**	**Group**	***n***	Females*	Age (years)*	Follow-up (months)*	History of high-risk comorbidities*†	**Living in rural areas**	**Previous use of antidiabetics**	**Elixhauser comorbidity score**	**Concurrent use of >4 drugs**	**Concurrent use of interfering drugs‡**
#1 ACEIs/ARBs plus NSAIDs	Cases	1687	886 (52.5)	81.5 [7.7]	2.7 [1.8]	670 (39.7)	312 (18.5)	433 (25.7)	3.8 [6.0]	1168 (69.2)	1029 (61.0)
	Controls	15 968	8389 (52.5)	81.4 [7.8]	2.6 [1.8]	6325 (39.6)	3096 (19.4)	2937 (18.4)	2.5 [4.8]	9465 (59.3)	7272 (45.5)
#2 ACEIs/ARBs or diuretics plus glucocorticoids	Cases	1993	1047 (52.5)	82.3 [7.7]	2.7 [1.8]	945 (47.4)	378 (19.0)	491 (24.6)	5.3 [7.0]	1372 (68.8)	1247 (62.6)
	Controls	18 762	9822 (52.4)	82.1 [7.8]	2.6 [1.8]	8974 (47.8)	3735 (19.9)	3595 (19.2)	3.4 [5.7]	11 172 (59.5)	8723 (46.5)
#3 Diuretics plus NSAIDs	Cases	1101	570 (51.8)	83.7 [7.4]	2.3 [1.8]	696 (63.2)	212 (19.3)	289 (26.3)	8.1 [7.5]	718 (65.2)	962 (87.4)
	Controls	9194	4661 (50.7)	83.6 [7.5]	1.9 [1.6]	6050 (65.8)	1899 (20.7)	2179 (23.7)	6.5 [6.8]	5438 (59.1)	7373 (80.2)
#4 SSRIs plus NSAIDs	Cases	69	44 (63.8)	82.2 [7.4]	2.6 [1.8]	27 (39.1)	18 (26.1)	13 (18.8)	3.9 [7.2]	47 (68.1)	50 (72.5)
	Controls	638	404 (63.3)	82.2 [7.1]	2.5 [1.7]	253 (39.7)	134 (21.0)	107 (16.8)	2.2 [4.8]	374 (58.6)	360 (56.4)
#5 Vitamin K antagonists plus NSAIDs	Cases	61	31 (50.8)	82.5 [6.5]	2.5 [1.8]	21 (34.4)	5 (8.2)	15 (24.6)	5.4 [6.3]	27 (44.3)	24 (39.3)
	Controls	478	252 (52.7)	82.4 [6.5]	2.1 [1.6]	167 (34.9)	77 (16.1)	97 (20.3)	5.1 [6.6]	227 (47.5)	151 (31.6)
#6 NOACs plus NSAIDs	Cases	28	15 (53.6)	81.8 [6.0]	2.6 [1.7]	11 (39.3)	8 (28.6)	5 (17.9)	4.6 [6.1]	11 (39.3)	9 (32.1)
	Controls	263	139 (52.9)	82.0 [5.8]	2.5 [1.6]	105 (39.9)	44 (16.7)	34 (12.9)	4.1 [5.9]	106 (40.3)	51 (19.4)
#7 Vitamin K antagonists plus antibiotics/antimycotics	Cases	61	31 (50.8)	82.5 [6.5]	2.5 [1.8]	21 (34.4)	5 (8.2)	15 (24.6)	5.4 [6.3]	27 (44.3)	24 (39.3)
	Controls	499	258 (51.7)	82.5 [6.7]	2.2 [1.6]	173 (34.7)	101 (20.2)	83 (16.6)	4.8 [6.5]	237 (47.5)	157 (31.5)
#8 Antihypertensives plus α-blockers	Cases	1407	934 (66.4)	81.9 [7.8]	2.9 [1.8]	86 (6.1)	245 (17.4)	263 (18.7)	3.1 [5.8]	932 (66.2)	635 (45.1)
	Controls	13 112	8695 (66.3)	81.7 [7.8]	2.8 [1.8]	789 (6.0)	2570 (19.6)	2065 (15.7)	1.8 [4.4]	7996 (61.0)	3766 (28.7)
#9 Antidiabetics plus fluoroquinolones	Cases	110	50 (45.5)	78.7 [8.1]	2.6 [1.7]	-	24 (21.8)	93 (84.6)	4.1 [6.4]	65 (59.1)	48 (43.6)
	Controls	942	434 (46.1)	78.6 [7.9]	2.4 [1.6]	208 (22.1)	870 (92.4)	1.9 [4.6]	524 (55.6)	475 (50.4)
#10 SSRIs plus ASA	Cases	69	44 (63.8)	82.2 [7.4]	2.6 [1.8]	27 (39.1)	18 (26.1)	13 (18.8)	3.9 [7.2]	47 (68.1)	30 (43.5)
	Controls	622	395 (63.5)	82.2 [7.2]	2.4 [1.7]	247 (39.7)	126 (20.3)	108 (17.4)	2.8 [5.4]	328 (52.7)	198 (31.8)

* Matching variable.

† Hypertensive crisis, acute myocardial infarction, heart failure or kidney failure (analyses #1-3), hypertensive crisis, cerebrovascular event, intracranial bleeding, gastrointestinal bleeding or other hemorrhagic diathesis (analyses #4-7, #10), syncope or orthostatic hypotension (analysis #8).

‡ Interfering medications are listed in Supplementary Table 2.

**Figure 1:**
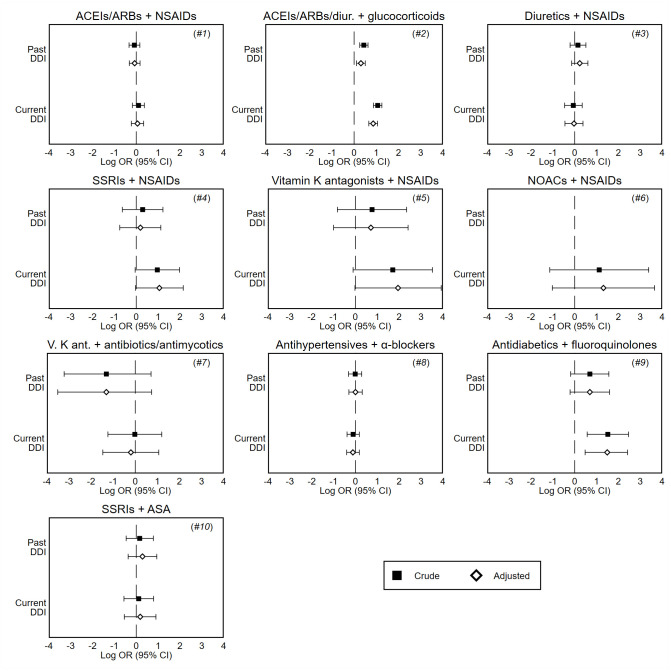
**Forest plots of crude and adjusted odds ratios of hospitalization associated with current (last month) and past (≥2 months before) exposure to DDI, by interaction analysis.** These odds ratios are unbiased estimates of the relative risk of hospitalization compared to no exposure to DDI, and are presented on the log scale. *Note:* Odds ratios are adjusted for covariates shown in Table 2. *DDI*, drug-drug interaction; *CI*, confidence interval; *ACEIs*, angiotensin converting enzyme inhibitors; *ARBs*, angiotensin II receptor blockers; *SSRIs*, selective serotonin reuptake inhibitors; *NOACs*, novel oral anticoagulants; *NSAIDs*, nonsteroidal anti-inflammatory drugs; *ASA*, acetylsalicylic acid.

Table 3 and Figure 1 show the results of the primary analysis. After adjusting for potentially confounding factors, we found that the combination of antihypertensive therapy (ACEIs/ARBs or diuretics) and glucocorticoids (analysis #2) was associated with an increased risk of hospitalization. This association reached statistical significance for both current (adj. OR 2.36; 95% CI 1.94-2.87; *P* <0,001) and past users (adj. OR 1.36; 95% CI 1.12-1.65; *P* 0.002). The vast majority of these hospitalizations were due to cardiovascular diseases (37.5% heart failure, 32.5% cerebrovascular events, 12.0% AMI, 5.8% hypertensive crisis), while the remaining ones were due to acute kidney failure (10.6%) and hyponatremia (1.7%). We also found an increased risk of hospitalization among current users of antidiabetics and fluoroquinolones (analysis #9: adj. OR 4.43; 95% CI 1.61-11.2; *P* 0.003); complications of diabetes accounted for the most hospitalizations (90.9%), followed by hypoglycemic coma (9.1%). In analysis #4 (SSRIs plus NSAIDs) and #5 (vitamin K antagonists plus NSAIDs) current users showed an increased risk, but failed to achieve statistical significance (analysis #4: adj. OR 2.88, 95% CI 0.97-8.59; analysis #5: adj. OR 7.01, 95% CI 0.98-50.4). These two interaction analyses had limited statistical power due to the low number of cases exposed to DDIs, as also confirmed by the large minimum detectable effect sizes (analysis #4: OR 3.92; analysis #5: OR 7.61).

**Table 3:** Odds ratios of hospitalization associated with current (last month) and past (≥2 months before) exposure to DDI.

**# Interaction analysis**	**Exposure to DDI**	**Cases**	**Matched controls**	**OR (95% CI)**		**Minimum detectable OR†**
				**Crude**	Adjusted*	
#1 ACEIs/ARBs plus NSAIDs	No	1549 (91.8)	14 676 (91.9)	Ref.	Ref.	Ref.
	Past	78 (4.6)	776 (4.9)	0.92 (0.72-1.18)	0.93 (0.73-1.19)	1.36
	Current	60 (3.6)	516 (3.2)	1.11 (0.84-1.45)	1.06 (0.80-1.40)	1.44
#2 ACEIs/ARBs or diuretics plus glucocorticoids	No	1698 (85.2)	17 299 (92.2)	Ref.	Ref.	Ref.
	Past	142 (7.1)	904 (4.8)	1.55‡ (1.29-1.87)	1.36‡ (1.12-1.65)	1.34
	Current	153 (7.7)	559 (3.0)	2.89‡ (2.39-3.49)	2.36‡ (1.94-2.87)	1.42
#3 Diuretics plus NSAIDs	No	1038 (94.3)	8734 (95.0)	Ref.	Ref.	Ref.
	Past	36 (3.3)	228 (2.5)	1.16 (0.81-1.67)	1.26 (0.87-1.83)	1.63
	Current	27 (2.5)	232 (2.5)	0.94 (0.63-1.41)	0.97 (0.64-1.46)	1.63
#4 SSRIs plus NSAIDs	No	58 (84.1)	577 (90.4)	Ref.	Ref.	Ref.
	Past	6 (8.7)	42 (6.6)	1.34 (0.53-3.39)	1.21 (0.47-3.08)	2.94
	Current	5 (7.2)	19 (3.0)	2.62 (0.95-7.22)	2.88 (0.97-8.59)	3.92
#5 Vitamin K antagonists plus NSAIDs	No	57 (93.4)	467 (97.7)	Ref.	Ref.	Ref.
	Past	2 (3.3)	7 (1.5)	2.14 (0.44-10.4)	2.03 (0.37-11.1)	5.73
	Current	2 (3.3)	4 (0.8)	5.52 (0.90-33.8)	7.01 (0.98-50.4)	7.61
#6 NOACs plus NSAIDs	No	27 (96.4)	255 (97.0)	Ref.	Ref.	Ref.
	Past	0 (0.0)	5 (1.9)	n/a	n/a	7.86
	Current	1 (3.6)	3 (1.1)	3.07 (0.32-29.5)	3.72 (0.36-38.6)	10.0
#7 Vitamin K antagonists plus antibiotics or antimycotics	No	57 (93.4)	449 (90.0)	Ref.	Ref.	Ref.
	Past	1 (1.6)	25 (5.0)	0.27 (0.04-2.04)	0.27 (0.03-2.09)	3.35
	Current	3 (4.9)	25 (5.0)	0.98 (0.29-3.31)	0.82 (0.23-2.89)	3.30
#8 Antihypertensives plus α-blockers	No	1289 (91.6)	11 958 (91.2)	Ref.	Ref.	Ref.
	Past	54 (3.8)	498 (3.8)	0.99 (0.73-1.33)	1.00 (0.74-1.36)	1.44
	Current	64 (4.5)	656 (5.0)	0.90 (0.68-1.19)	0.89 (0.67-1.19)	1.39
#9 Antidiabetics plus fluoroquinolones	No	96 (87.3)	896 (95.1)	Ref.	Ref.	Ref.
	Past	7 (6.4)	31 (3.3)	2.00 (0.83-4.77)	2.00 (0.81-4.90)	3.23
	Current	7 (6.4)	15 (1.6)	4.54‡ (1.77-11.7)	4.43‡ (1.61-11.2)	4.40
#10 SSRIs plus ASA	No	42 (60.9)	400 (64.3)	Ref.	Ref.	Ref.
	Past	15 (21.7)	119 (19.1)	1.17 (0.63-2.19)	1.33 (0.69-2.56)	2.27
	Current	12 (17.4)	103 (16.6)	1.12 (0.57-2.21)	1.20 (0.58-2.46)	2.36

These odds ratios are unbiased estimates of the relative risk of hospitalization. Values are counts (percentages) unless stated otherwise.

* Adjusted for covariates shown in Table 2.

† The minimum detectable odds ratio (rate ratio) is the smallest effect that yields a statistically significant result for a pre-specified power (80%), sample size, and exposure probability among controls.

‡ Significant at the 0.05 level or less.

*OR*, odds ratio; *CI*, confidence interval.

**Figure 2:**
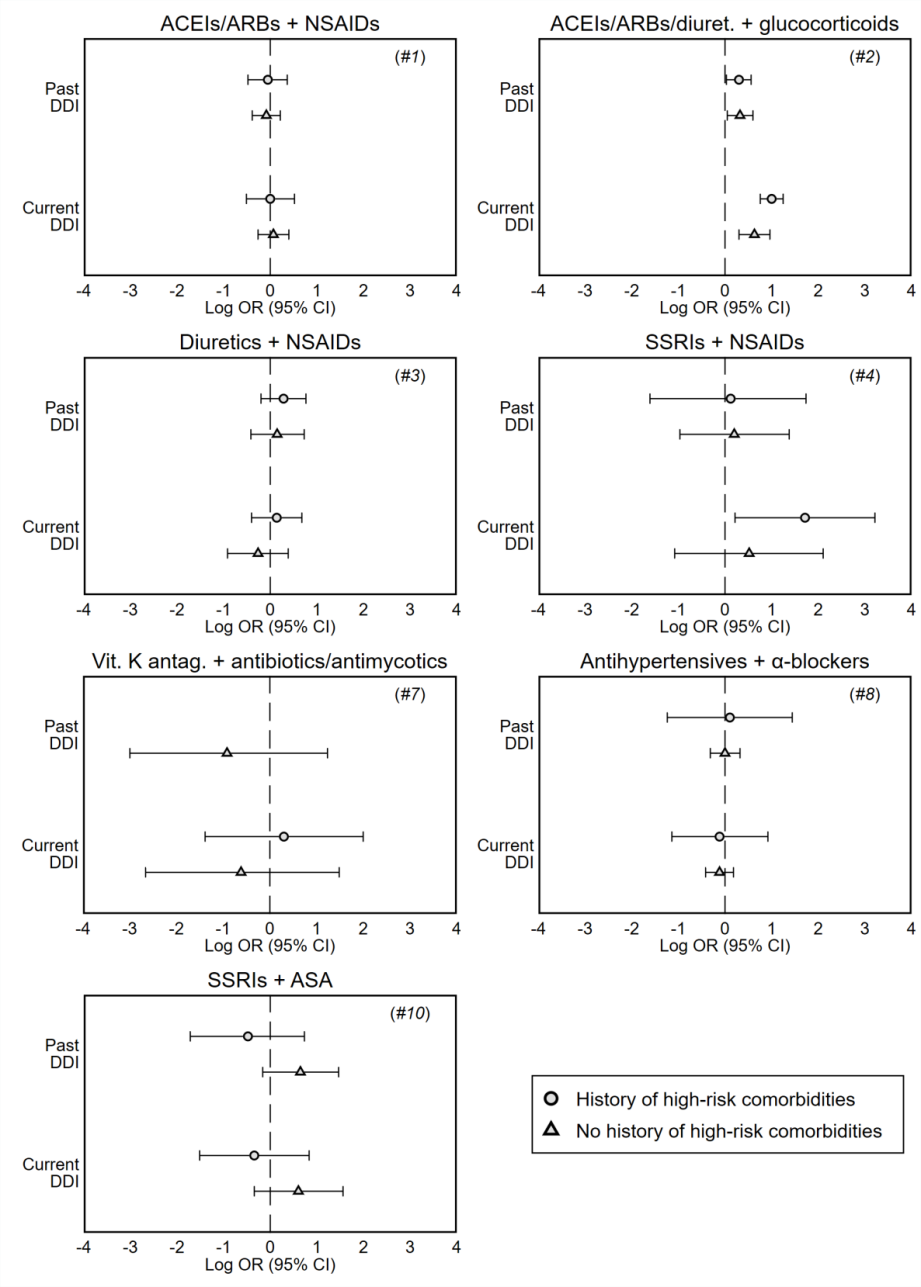
**Forest plots of adjusted odds ratios of hospitalization associated with current (last month) and past (≥2 months before) exposure to DDI, stratified by history of high-risk comorbidities in the previous 3 years (see Supplementary Table 3).** These odds ratios are unbiased estimates of the relative risk of hospitalization compared to no exposure to DDI, and are presented on the log scale. Analyses #5 and #6 are not presented due to the limited number of patients exposed to DDI per stratum; history of high-risk comorbidities was not investigated in analysis #9. *Note:* Odds ratios are adjusted for covariates shown in Table 2.

When we stratified cases and matched controls by the presence of hospitalizations for high-risk conditions in the previous 3-year period, the results were generally consistent with those of the primary analysis, with the exception of analysis #4 (Table 4 and Figure 2). Current use of SSRIs plus NSAIDs was significantly associated with an increased risk of hospitalization for patients who had a history of disease (adj. OR 5.56; 95% CI 1.24-24.9; P 0.025).

**Table 4:** Odds ratios of hospitalization associated with current (last month) and past (≥2 months before) exposure to DDI, stratified by history of high-risk comorbidities in the previous 3 years (see Supplementary Table 3).

**Interaction analysis**	**Exposure to DDI**	**History of high-risk comorbidities**				**No history of high-risk comorbidities**			
		**Cases**	**Matched controls**	**OR (95% CI)**		**Cases**	**Matched controls**	**OR (95% CI)**	
				**Crude**	Adjusted*			**Crude**	Adjusted*
#1 ACEIs/ARBs plus NSAIDs	No	627 (93.6)	5882 (93.0)	Ref.	Ref.	922 (90.7)	8794 (91.2)	Ref.	Ref.
	Past	26 (3.9)	270 (4.3)	0.88 (0.58-1.33)	0.95 (0.62-1.44)	52 (5.1)	506 (5.2)	0.95 (0.70-1.28)	0.92 (0.68-1.24)
	Current	17 (2.5)	173 (2.7)	0.93 (0.56-1.54)	1.00 (0.60-1.68)	43 (4.2)	343 (3.6)	1.20 (0.87-1.66)	1.07 (0.77-1.49)
#2 ACEIs/ARBs or diuretics plus glucocorticoids	No	766 (81.1)	8127 (90.6)	Ref.	Ref.	932 (88.9)	9172 (93.7)	Ref.	Ref.
	Past	75 (7.9)	499 (5.6)	1.53† (1.18-1.98)	1.35† (1.03-1.75)	67 (6.4)	405 (4.1)	1.58† (1.21-2.08)	1.38† (1.05-1.82)
	Current	104 (11.0)	348 (3.9)	3.28† (2.59-4.14)	2.72† (2.13-3.48)	49 (4.7)	211 (2.2)	2.33† (1.69-3.21)	1.88† (1.35-2.62)
#3 Diuretics plus NSAIDs	No	659 (94.7)	5784 (95.6)	Ref.	Ref.	379 (93.6)	2950 (93.8)	Ref.	Ref.
	Past	21 (3.0)	136 (2.2)	1.19 (0.74-1.91)	1.33 (0.82-2.15)	15 (3.7)	92 (2.9)	1.11 (0.63-1.97)	1.16 (0.66-2.07)
	Current	16 (2.3)	130 (2.2)	1.06 (0.63-1.80)	1.15 (0.67-1.97)	11 (2.7)	102 (3.2)	0.80 (0.42-1.51)	0.77 (0.40-1.47)
#4 SSRIs plus NSAIDs	No	22 (81.5)	230 (90.9)	Ref.	Ref.	36 (85.7)	347 (90.1)	Ref.	Ref.
	Past	2 (7.4)	15 (5.9)	1.30 (0.27-6.26)	1.13 (0.20-5.68)	4 (9.5)	27 (7.0)	1.37 (0.43-4.34)	1.22 (0.38-3.96)
	Current	3 (11.1)	8 (3.2)	3.92 (0.96-16.0)	5.56† (1.24-24.9)	2 (4.8)	11 (2.9)	1.77 (0.39-8.15)	1.68 (0.34-8.21)
#7 Vitamin K antagonists plus antibiotics or antimycotics	No	19 (90.5)	152 (87.9)	Ref.	Ref.	38 (95.0)	297 (91.1)	Ref.	Ref.
	Past	0 (0.0)	10 (5.8)	n/a	n/a	1 (2.5)	15 (4.6)	0.45 (0.06-3.61)	0.40 (0.05-3.43)
	Current	2 (9.5)	11 (6.4)	1.50 (0.32-7.08)	1.35 (0.25-7.37)	1 (2.5)	14 (4.3)	0.58 (0.07-4.50)	0.54 (0.07-4.41)
#8 Antihypertensives plus α-blockers	No	78 (90.7)	705 (89.4)	Ref.	Ref.	1211 (91.7)	11 253 (91.3)	Ref.	Ref.
	Past	3 (3.5)	31 (3.9)	0.82 (0.22-3.00)	1.11 (0.29-4.23)	51 (3.9)	467 (3.8)	1.00 (0.73-1.36)	1.00 (0.73-1.38)
	Current	5 (5.8)	53 (6.7)	0.82 (0.30-2.25)	0.89 (0.32-2.51)	59 (4.5)	603 (4.9)	0.91 (0.68-1.22)	0.89 (0.66-1.20)
#10 SSRIs plus ASA	No	19 (70.4)	141 (57.1)	Ref.	Ref.	23 (54.8)	259 (69.1)	Ref.	Ref.
	Past	4 (14.8)	56 (22.7)	0.53 (0.17-1.61)	0.62 (0.18-2.08)	11 (26.2)	63 (16.8)	1.90 (0.88-4.14)	1.91 (0.85-4.33)
	Current	4 (14.8)	50 (20.2)	0.59 (0.19-1.81)	0.71 (0.22-2.30)	8 (19.0)	53 (14.1)	1.76 (0.74-4.19)	1.83 (0.71-4.76)

These odds ratios are unbiased estimates of the relative risk of hospitalization. Values are counts (percentages) unless stated otherwise. Analyses #5 and #6 are not presented due to the limited number of patients exposed to DDI per stratum; history of high-risk comorbidities was not investigated in analysis #9.

* Adjusted for covariates shown in Table 2.

† Significant at the 0.05 level or less.

### Sensitivity analyses

When we adjusted the models for prevalent user status, the results were virtually coincident with those of the primary analysis (Supplementary Table 4); the combination of ACEIs/ARBs or diuretics and glucocorticoids was significantly associated with an increased risk of hospitalization (past use: adj. OR 1.36, 95% CI 1.12-1.64, *P* 0.002; current use: adj. OR 2.35, 95% CI 1.93-2.86, *P* <0.001).

When we examined whether DDIs were associated with an increased risk of either hospitalization or specialist examination/consultation, whichever occurred first, results were not fully consistent with those of the primary analysis (Table 5). The directions of the odds (risks) changed for analysis #1 (ACEIs/ARBs plus NSAIDs), #3 (diuretics plus NSAIDs), #5 (vitamin K antagonists plus NSAIDs), #6 (NOACs plus NSAIDs), #7 (vitamin K antagonists plus antibiotics/antimycotics) and #10 (SSRIs plus ASA). However, the increased risk associated with taking ACEIs/ARBs or diuretics plus glucocorticoids (adj. OR 1.95; 95% CI 1.72-2.20; *P* <0.001) and antidiabetics plus fluoroquinolones (adj. OR 1.52; 95% CI 1.23-1.89; *P* <0.001) was confirmed.

**Table 5:** Odds ratios of hospitalization/specialist examination (see Supplementary Table 1) associated with current (last month) and past (≥2 months before) exposure to DDI.

**# Interaction analysis**	**Exposure to DDI**	**Cases**	**Matched controls**	**OR (95% CI)**		**Minimum detectable OR†**
				**Crude**	Adjusted*	
#1 ACEIs/ARBs plus NSAIDs	No	4396 (91.6)	41 902 (91.5)	Ref.	Ref.	Ref.
	Past	243 (5.1)	2328 (5.1)	0.96 (0.84-1.11)	0.91 (0.79-1.05)	1.20
	Current	161 (3.3)	1571 (3.4)	0.98 (0.83-1.15)	0.89 (0.75-1.05)	1.24
#2 ACEIs/ARBs or diuretics plus glucocorticoids	No	4868 (87.0)	49 010 (92.6)	Ref.	Ref.	Ref.
	Past	359 (6.4)	2314 (4.4)	1.54‡ (1.37-1.73)	1.35‡ (1.20-1.52)	1.20
	Current	370 (6.6)	1585 (3.0)	2.39‡ (2.12-2.68)	1.95‡ (1.72-2.20)	1.24
#3 Diuretics plus NSAIDs	No	2212 (94.6)	17 908 (94.6)	Ref.	Ref.	Ref.
	Past	72 (3.1)	547 (2.9)	0.90 (0.70-1.16)	0.91 (0.70-1.18)	1.39
	Current	53 (2.3)	477 (2.5)	0.90 (0.67-1.20)	0.89 (0.66-1.19)	1.42
#4 SSRIs plus NSAIDs	No	1140 (88.6)	10 522 (90.6)	Ref.	Ref.	Ref.
	Past	83 (6.4)	638 (5.5)	1.14 (0.89-1.45)	1.12 (0.88-1.43)	1.39
	Current	64 (5.0)	458 (3.9)	1.28 (0.98-1.68)	1.24 (0.95-1.63)	1.46
#5 Vitamin K antagonists plus NSAIDs	No	416 (97.2)	3376 (96.2)	Ref.	Ref.	Ref.
	Past	6 (1.4)	71 (2.0)	0.58 (0.25-1.36)	0.60 (0.25-1.42)	2.18
	Current	6 (1.4)	64 (1.8)	0.73 (0.31-1.70)	0.69 (0.29-1.62)	2.25
#6 NOACs plus NSAIDs	No	364 (96.5)	3404 (95.5)	Ref.	Ref.	Ref.
	Past	9 (2.4)	106 (3.0)	0.76 (0.38-1.52)	0.76 (0.38-1.52)	2.03
	Current	4 (1.1)	53 (1.5)	0.69 (0.25-1.91)	0.66 (0.24-1.83)	2.51
#7 Vitamin K antagonists plus antibiotics or antimycotics	No	401 (90.3)	3412 (93.3)	Ref.	Ref.	Ref.
	Past	24 (5.4)	152 (4.2)	1.14 (0.73-1.79)	1.06 (0.67-1.67)	2.03
	Current	19 (4.3)	94 (2.6)	1.79 (0.97-3.04)	1.67 (0.99-2.81)	2.13
#9 Antidiabetics plus fluoroquinolones	No	3634 (92.9)	30 728 (94.3)	Ref.	Ref.	Ref.
	Past	174 (4.5)	1231 (3.8)	1.08 (0.91-1.27)	1.16 (0.98-1.36)	1.26
	Current	103 (2.6)	619 (1.9)	1.42‡ (1.15-1.75)	1.52‡ (1.23-1.89)	1.38
#10 SSRIs plus ASA	No	885 (70.2)	7751 (68.4)	Ref.	Ref.	Ref.
	Past	204 (16.2)	1802 (15.9)	0.97 (0.82-1.14)	1.01 (0.86-1.20)	1.24
	Current	171 (13.6)	1774 (15.7)	0.84 (0.71-1.00)	0.87 (0.73-1.04)	1.25

These odds ratios are unbiased estimates of the relative risk. Values are counts (percentages) unless stated otherwise.

* Adjusted for covariates shown in Table 2.

† The minimum detectable odds ratio (rate ratio) is the smallest effect that yields a statistically significant result for a pre-specified power (80%), sample size, and exposure probability among controls.

‡ Significant at the 0.05 level.

Considering patients who had been hospitalized for specific conditions as exposed to NSAIDs even in the absence of recent reimbursed prescriptions, results did not change appreciably and, again, the increased risk associated with concurrent use of SSRIs and vitamin K antagonists (analyses #4, #5) failed to achieve statistical significance (Table 6).

**Table 6:** Odds ratios of hospitalization associated with current (last month) and past (≥2 months before) exposure to NSAIDs.

**# Interaction analysis**	**Exposure to DDI**	**Cases**	**Matched controls**	**OR (95% CI)**		**Minimum detectable OR†**
				**Crude**	Adjusted*	
#1 ACEIs/ARBs plus NSAIDs	No	1507 (89.3)	14 344 (89.8)	Ref.	Ref.	Ref.
	Past	79 (4.7)	774(4.8)	0.94 (0.74-1.20)	0.94 (0.74-1.21)	1.36
	Current	101 (6.0)	850 (5.3)	1.13 (0.92-1.41)	1.13 (0.91-1.40)	1.34
#3 Diuretics plus NSAIDs	No	1009 (91.6)	8496 (92.4)	Ref.	Ref.	Ref.
	Past	38 (3.5)	234 (2.5)	1.20 (0.84-1.72)	1.31 (0.91-1.87)	1.62
	Current	54 (4.9)	464 (5.0)	0.98 (0.73-1.31)	1.01 (0.75-1.36)	1.44
#4 SSRIs plus NSAIDs	No	56 (81.2)	558 (87.5)	Ref.	Ref.	Ref.
	Past	6 (8.7)	39 (6.1)	1.48 (0.58-3.75)	1.37 (0.53-3.51)	3.02
	Current	7 (10.1)	41 (6.4)	1.73 (0.74-4.01)	2.08 (0.86-5.01)	2.95
#5 Vitamin K antagonists plus NSAIDs	No	54 (88.5)	454 (95.0)	Ref.	Ref.	Ref.
	Past	2 (3.3)	7 (1.5)	2.21 (0.45-10.8)	2.07 (0.41-10.5)	5.82
	Current	5 (8.2)	17 (3.6)	2.64 (0.93-7.51)	2.55 (0.90-7.25)	3.84
#6 NOACs plus NSAIDs	No	27 (96.4)	244 (92.8)	Ref.	Ref.	Ref.
	Past	0 (0.0)	5 (1.9)	n/a	n/a	7.68
	Current	1 (3.6)	14 (5.3)	0.67 (0.08-5.31)	0.70 (0.08-6.06)	4.76

These odds ratios are unbiased estimates of the relative risk. Values are counts (percentages) unless stated otherwise. Patients exposed to DDI are those who had a filled prescription for NSAIDs or went to the hospital for a condition indicating NSAID use (see Supplementary Table 4).

* Adjusted for covariates shown in Table 3.

† The minimum detectable odds ratio (rate ratio) is the smallest effect that yields a statistically significant result for a pre-specified power (80%), sample size, and exposure probability among controls.

Lastly, to account for immeasurable time bias, we restricted the analyses to cases and controls who spent <50% of their matched follow-up periods in the hospital. The results of this sensitivity analysis were virtually coincident with those of the primary analysis (Supplementary Table 6).

## DISCUSSION

In the large population assessed in this study, we found that the current use of glucocorticoids was associated with a 2.36-fold increase in the risk of hospitalization for cardiovascular diseases or acute kidney failure among older patients under ACE-inhibitors, angiotensin-receptor blockers or diuretics. An increased risk also emerged for patients who had used glucocorticoids in the past, although it was lower (1.36-fold). This risk was even higher in patients with high-risk conditions, although statistical significance was maintained in the low-risk population.

Additionally, we found a significant association between current use of fluoroquinolones and hospitalization for diabetic complications in patients taking antidiabetic drugs (4.54-fold).

In patients with SSRI therapy and a history of bleeding episodes, current use of NSAIDs showed a 5.56-fold increase risk ratio of recurrence of bleeding.

High-risk conditions led to a small increase in the risk ratio for a few DDIs. In contrast to our hypothesis, this phenomenon was not shown for all DDIs; as a matter of fact, fewer prescriptions of NSAIDs occurred in patients taking ACEI/ARBs or diuretics (Table 4, analyses #1 and #3), suggesting that physicians are cautious in prescribing NSAIDs when the patient has a history of acute kidney injury or cardiovascular disease.

We did not find a significantly increased risk ratio for all the other potential DDIs, suggesting that they have only minimal clinical consequences, at least within the pattern of drug use in the older population of Bologna’s area (for instance, NSAIDs seem safe in patients treated with ACEIs/ARBs).

When we added data on specialized visits and imaging to our outcome definition (such as nephrological visits), the direction of odds changed in most of the DDIs although none of these effect sizes was statistically significant. However, since most of the chronic disorders (e.g. diabetes) need strict monitoring of the trend of disease and the benefit-risk profile of drug therapies, increasing visits may be proof of increasing care rather than adverse outcome.

### ACE-inhibitors (or diuretics) plus glucocorticoids

The increased risk of cardiovascular diseases in patients taking glucocorticoids is well known (at least 25%), as well as its dose-dependence [10–15]. It should first be recognized that the activity of inflammatory disease in these patients (e.g. with rheumatoid arthritis) can increase *per se* the cardiovascular risk, thereby representing a confounder by indication [10]. Glucocorticoid mechanism of action is complex and involves elevated angiotensinogen synthesis, increased sympathetic nervous system activation, and mineralocorticoid-like action. Also the weight gain and the android fat distribution due to glucocorticoids seem to contribute [16]*.*

As for DDI with ACEIs/ARBs or diuretics, glucocorticoids can antagonize their effect and cause a loss of blood pressure control, thus secondary hypertension and pseudo-resistant hypertension [17]. Patients with a history of essential hypertension are certainly at greater risk of developing secondary hypertension due to from glucocorticoids and relevant cardiovascular diseases [16], and in fact the risk ratio found in our study was 2.36. The cumulative effect of glucocorticoids could also explain the still detectable, although lower, risk in patients with past exposure to glucocorticoids. As regards the mean age of our cases (82 years), it is in line with the higher risk of older patients to developing glucocorticoid-induced hypertension [10–18]. Patients taking glucocorticoids are even more likely to develop other well-known cardiovascular risk factors, such as diabetes mellitus and hypercholesterolemia [16]. The direct effects of the glucocorticoid receptor on heart and blood vessels affect vascular function, remodeling and atherogenesis, and therefore also contribute to cardiovascular diseases [19].

It is not possible to completely avoid glucocorticoids in patients with ACEIs/ARBs or diuretic therapy, since they are highly effective and sometimes crucial for diseases that require immediate suppression of inflammation and immune activity, such as chronic obstructive pulmonary disease or rheumatoid arthritis. However, taking into account the results of this study, blood pressure monitoring is strongly recommended during glucocorticoid therapy, together with reduction in the length of glucocorticoid cycles.

### Antidiabetics plus fluoroquinolones

Our results are in line with previous studies that show an association between the use of fluoroquinolones and dysglycemia (hypo- or hyperglycemia) compared to other antibacterial (macrolides). Gatifloxacin was even withdrawn from the market, while levofloxacin, ciprofloxacin and moxifloxacin are maintained on the market despite association with dysglycemia, as their benefit-risk profile remained favorable [20–22]. Fluoroquinolones are more frequently associated with hyperglycemia [23], although hypoglycemia can also lead to severe cases and even fatal outcomes [24]. We must acknowledge that infections in patients with diabetes are per se a common cause of dysglycemia. The high risk-ratio of 4.4 in the current study could be partly due to this effect, since we studied the effect of fluoroquinolones compared to absence of fluoroquinolones in patients taking antidiabetic drugs. On the other hand, the interaction with antidiabetic drugs can independently contribute to dysglycemia.

A recent analysis of the FDA Adverse Event Reporting System *(*FAERS) on reports of hypoglycemia for various antimicrobial therapies also found an increased reporting of hypoglycemia in patients using fluoroquinolones (reporting odds ratio=1.6).

Inhibition of potassium-ATP(K-ATP) channels in pancreatic B-cells, with consequent insulin secretion increase [25], and inhibition of antidiabetic CYP metabolism by fluoroquinolones [26] have been postulated as mechanisms of hypoglycemia. Instead, mechanism of the more common fluoroquinolone-induced hyperglycemia is to date unknown.

When antibiotic therapy is advised in patients with antidiabetic treatment, it is recommended to consider not only the antimicrobial potency of the antibiotics, but also the risk for potentially serious adverse effects. Albeit rare, fluoroquinolone-induced dysglycemia may be serious, so these medications are not recommended for this group of patients.

### SSRIs plus NSAIDs

Increased bleeding risk for SSRIs plus NSAIDs is well described in the literature [27–30]. They both have antiplatelet activity, thereby they have a synergistic effect on hemostatic function. In the current study, we failed to achieve a statistically significant association between bleeding and use of NSAIDs in patients under SSRIs, although the risk ratio was about 3.

Instead, in patients with a previous episode of bleeding, we found a significant 5.6-fold risk ratio of bleeding in patients currently using NSAIDs concomitantly with SSRIs, leading us to conclude that patients with SSRI therapy and previous bleeding have a higher risk of recurrent bleeding when using NSAID. These results are in line with a previous retrospective study [31] and strongly suggest the importance of stringent assessment of both benefit-risk profile and appropriateness for each individual patient before prescribing SSRIs and NSAIDs.

### History of comorbidities

Usually, previous comorbidities increase the risk of adverse events. However, in this study we only found an increased risk ratio for ACE-inhibitors/diuretics plus glucocorticoids and for SSRIs plus NSAIDs. This inconsistency could be due to the prescribing habits of physicians, as recently showed by Nash et al. [32], who found fewer NSAID prescriptions among patients with kidney injury or heart failure that receive long-term custodial care. However, it should be acknowledged that some interaction analyses had limited statistical power.

On the other hand, these findings might indicate that having a history of diseases does not increase the risk. Recent studies and systematic reviews have shown a similar risk for cardiovascular or renal diseases as a result of NSAID use in high-risk patients [33–35]. However, these studies only analyzed the use of NSAID, regardless of combination with other drugs, and the increased risk attributable to NSAIDs alone, if any, is probably too low to be detected in a cohort study.

### Strengths and limitations

We conducted a large population-based study, which to our knowledge is the first real-world evidence study on 10 different potential DDISs that also takes into account the role of high-risk comorbidities. Methodological strengths include the study design, in which cases and controls are matched by follow-up duration, thereby preventing time-related bias [36]*.* In addition, drug exposure was collected from the OPD, avoiding possible recall bias [37].

As for chronic drugs of interest, we used the threshold of 80% in the MPR as a proxy of continuous use. We chose to lower this threshold for SSRIs and NOAC to 66% due to very high variability in average daily doses and above all probable lower dose than the DDD in older populations. This approach could have influenced the results, by overestimating the number of patients exposed to DDIs in both current- and past-user groups. Given the average time of follow up of 2.5 months, no strong impact is expected.

In addition, data from dispensing databases is subject to measurement error. A dispensed package does not indicate that the full package is consumed, which is mostly common for short-cycle drugs or drugs taken on an as-needed basis. Furthermore, the prescribed daily doses could differ from the DDDs, especially for glucocorticoids, and this might have affected the validity of our results. Unfortunately, administrative data do not allow differentiating between high- and low-dosage or between long- and short-term medication use.

The use of NSAIDs could be particularly underestimated by only considering recent reimbursed prescriptions; notably patients could use over-the-counter (OTC) NSAIDs or NSAIDs prescribed in the past.

By adding other diagnoses, specialist visits and imaging as a proxy for disease, or including patients with conditions indicating use of NSAID to the sensitivity analyses, we have tried to minimize the information bias of OTC NSAIDs and the possible underestimation of incidence of the outcome. previous research showed that OTC drugs likely contribute to a small amount of bias [38].

Lastly, we did not consider a wide range of high-risk comorbidities, and had no data on other patient characteristics that could influence the study outcome (e.g., imaging with contrast, smoking habits, lifestyle, blood pressure, indication for drugs or severity of diseases).

## CONCLUSIONS

Among drug prescriptions in the elderly population of Bologna’s area, our findings distinguished concomitant drug therapies with actual clinical consequences from other treatments that can be considered generally safe: out of 10 pairs of DDIs, clinical adverse events emerged for glucocorticoid use in patients using ACE-inhibitors or diuretics, and fluoroquinolone use in antidiabetic drug recipients. NSAIDs increased the risk of re-bleeding in patients with SSRI therapy and a previous bleeding episode.

Observed prescribing habits of clinicians reflect high awareness of potential interactions in patients at risk, especially for NSAID prescription in patients taking antihypertensives who have a history of acute kidney injury or cardiovascular diseases. However, strict monitoring of patients exposed to the most clinically important DDIs and deprescribing initiatives are strongly recommended.

Future studies based on different data sources should focus on other variables potentially affecting susceptibility to ADRs due to DDIs, such as lifestyle, smoking habits, or severity of comorbidities, to help clinicians assess benefit-risk properties in an individual setting.

## MATERIALS AND METHODS

### Setting and study population

The study population comprised residents of the Local Healthcare Authority of Bologna in Northern Italy (≈876,000 inhabitants), aged ≥65, who were prescribed one of the chronic drugs of interest between January 2017 and June 2017 (see left side of Table 7 for the detailed list of chronic drug therapies with ATC codes). For each subject, cohort entry was the date of a first dispensed prescription of a chronic drug over the 6-month recruitment period. All patients were followed up to 6 months after the cohort entry.

**Table 7:** Summary of the DDIs investigated in the study.

**#**	**Chronic drug**	**ATC code**	**Interacting drug**	**ATC code**
1	ACEIs/ARBs	C09	NSAIDs	M01A
2	ACEIs/ARBs	C09	Glucocorticoids	H02
	Thiazide diuretics	C03A		
	Loop diuretics	C03C		
3	*Diuretics*		NSAIDs	M01A
	Thiazide diuretics	C03A		
	Loop diuretics	C03C		
4	SSRIs	N06AB	NSAIDs	M01A
5	Vitamin K antagonists	B01AA	NSAIDs	M01A
6	*NOACs*		NSAIDs	M01A
	Dabigatran	B01AE07		
	Rivaroxaban	B01AF01		
	Apixaban	B01AF02		
7	Vitamin K antagonists	B01AA	*Antibiotics*	
			Macrolide	J01FA
			Fluoroquinolones	J01MA
			Antimycotics	J02
8	*Antihypertensives*		α-blockers	G04CA
	Diuretics	C03		
	β-blockers	C07		
	Calcium channel blockers	C08		
	ACEIs/ARBs	C09		
9	Antidiabetics	A10	Fluoroquinolones	J01MA
10	SSRIs	N06AB	ASA	B01AC06

Chronic drugs of interest and ATC codes are on the left side; interacting drugs and ATC codes are on the right side.

*DDI*, drug-drug interaction; *ATC*, Anatomical Therapeutic Chemical; *ACEIs*, angiotensin converting enzyme inhibitors; *ARBs*, angiotensin II receptor blockers; *SSRIs*, selective serotonin reuptake inhibitors; *NOACs*, novel oral anticoagulants; *NSAIDs*, nonsteroidal anti-inflammatory drugs; *ASA*, acetylsalicylic acid.

Data were retrieved from the Regional Health Authority Outpatient Pharmaceutical Database (OPD), which contains information on patients (unique identification number, sex and age), prescriptions (substance name, ATC code (*WHO Collaborating Centre for Drug Statistics Methodology, ATC classification index with DDDs, 2020. Oslo, Norway 2019*), brand name, date of prescription filling, number of unit doses and number of packages) and prescribers; it does not include the actual prescribed daily dose of the drug. The OPD includes drugs reimbursed by the healthcare system that are prescribed by the primary care physician or the specialist, or directly dispensed by the hospital pharmacies [39].

### Exposure to drug-drug interactions

The 10 DDIs considered in our study derive from the Italian experience on prevalence of potentially inappropriate prescriptions (see the right side of Table 7 for the detailed list of interacting drugs with ATC codes) [39–41]. Dispensed prescriptions of interacting drugs were retrieved from the OPD.

### Study outcomes

The outcome of this study was represented by hospital admission due to a condition potentially induced by DDI as the principal diagnosis (see Supplementary Table 1 for the detailed list of DDI-related hospital admissions with ICD-9-CM codes for each interaction analysis). Data were retrieved from the Hospital Discharge Records (HDRs) Database, which can be linked to the OPD using the unique patient identifier.

All-cause deaths within 6 months of cohort entry were considered as censored events (source: Regional Mortality Register Database).

### Potential confounders

Aiming to keep our study fully generalizable to older populations, we did not exclude patients with a history of high-risk comorbidities (e.g., patients taking angiotensin converting enzyme inhibitors [ACEIs] or angiotensin receptor blockers [ARBs] with previous hospital admission for kidney failure). Instead, we adjusted all analyses for the presence of previous hospitalizations for these conditions (see Supplementary Table 3) over a lookback period of 3 years before the cohort entry (source: HDRs).

Other variables we analyzed to reduce the potential source of confounding were:

- Sex;
- Age;
- Degree of urbanization of the municipality where the patient lived, classified as city, towns/suburbs or rural, using the Eurostat’s Degree of Urbanization (DEGURBA) classification system (revised definition, 2014);
- Use of antidiabetic drug therapies in the 6 months prior to cohort entry (as proxy of diabetes, representing a major cardiovascular risk factor); Elixhauser comorbidity score based on hospitalizations in the previous 3 years [42];
- Number of concurrent medications during follow-up (>4 dispensations of different chemical subgroups– IV level ATC codes);

Use of interfering medications during follow-up that are known to be associated with the outcomes (see Supplementary Table 2).

### Statistical analysis

Numerical variables were summarized as mean ± standard deviation; categorical variables were summarized as frequencies and percentages.

Association between exposure (taking interacting drugs) and outcomes (DDI-related hospitalizations) was assessed using a nested case-control design. In each interaction analysis, patients hospitalized during follow-up were defined as cases, and up to 10 controls were randomly selected and matched to each case by follow-up duration, age (5-year groups), sex and history of high-risk conditions (see Supplementary Table 3). An illustrative example of this technique, which is called “incidence density sampling”, is provided in Supplementary Figure 11. We chose this approach to ensure an equal time window for measuring DDI exposure in cases and controls.

To focus the analyses on adherent users, cases and matched controls were excluded if the proportion of days covered by chronic medications between cohort entry and the matching date was <80% (<66% for SSRIs [analyses #4, #10] and vitamin K antagonists [#5, #7]). The proportion of days covered was estimated by using the medication possession ratio (MPR), which was based on the defined daily doses (DDDs; *WHO Collaborating Centre for Drug Statistics Methodology, ATC classification index with DDDs, 2020. Oslo, Norway 2019*). The DDD represents the average adult dose used for the main indication of the drug and thereby allows approximate quantification of days supplied. We set a MPR of 80% as a proxy for chronic therapy of the drugs of interest, because the DDDs may be different from the prescribed daily doses, possibly leading to an underestimation of adherence.

Cases and controls were then classified into 3 mutually exclusive groups: (i) current exposure, (ii) past exposure and (iii) no exposure to DDI. A subject was considered currently exposed if the prescription of the interacting drug was detected in the 30-day period prior to matching date. The ‘past-exposed group’ included persons with the last prescription over 30 days before the matching date. Subjects who were not prescribed the interacting drug during the matched follow-up period were considered as non-exposed to DDI. Dispensed prescriptions of interaction drugs were also collected in the 2-month period prior to cohort entry: if a prescription was present and, on the basis of the number of DDDs contained in the packages, relevant doses also covered the days after the cohort entry, the patient was considered as exposed to DDI. We did this to mitigate possible underestimation in the number of prescriptions among cases and controls with short matched follow-up periods.

In a secondary analysis, we stratified cases and matched controls by the presence of previous hospitalizations for high-risk conditions (see Supplementary Table 3) to assess the effect of this variable in the main models (this was a matching variable). The association between DDI and outcomes was estimated using conditional logistic regression, which is appropriate for a time-matched nested case-control study. Results were expressed as odds ratios that, for the incidence density sampling used in this study, provide unbiased estimates of the relative risks (rate ratios) in the underlying cohort [43]. Regression models included all the potential confounders described above.

All analyses were carried out by using Stata software, version 15 (StataCorp. 2017. *Stata Statistical Software: Release 15.* College Station, TX: StataCorp LP). The significance level was set at 0.05.

### Sensitivity analyses

We conducted some sensitivity analyses to test the robustness of the findings from the primary analysis. First, we adjusted analyses by prevalent versus incident user status (presence versus absence of other dispensed prescriptions for the chronic drug of interest in the 6 months prior to cohort entry) to assess the potential confounding effect of being a prevalent or incident medication user. Second, we added information from the Outpatient Care Database to the outcomes to additionally assess the effect of including information on specialist visits and (non-)invasive imaging (see the codes in italics in Supplementary Table 1). Third, in an attempt to capture over-the-counter medications, we considered as NSAID users (either past or current) those patients who had been hospitalized for diseases that indicate NSAID use (Supplementary Table 5). Last, we excluded patients who spent >50% of their individual follow-up in the hospital, because drugs dispensed during inpatient treatment cannot be retrieved from the OPD, possibly leading to immeasurable time bias [44].

### Ethics statement

Ethical approval was granted from the *Comitato Etico di Area Vasta Emilia Centro* (Submission Number 611/2019/OSS/AUSLBO). This retrospective study was carried out in conformity with the regulations on data management with the Italian law on privacy (Legislation Decree 196/2003 amended by Legislation Decree 101/2018).

### Impact statement

We certify that this work is original research. It identified some specific drug-drug interactions with actual increased risk of hospitalization: ACE-inhibitors (or diuretics) plus glucocorticoids, antidiabetics plus fluoroquinolones, SSRIs plus NSAIDs in patients with previous bleeding episodes. Many other potential drug-drug interactions with high prevalence (for instance, ACE-inhibitors plus NSAIDs) did not seem to have an impact on adverse clinical outcomes.

## Supplementary Material

Supplementary Figures

Supplementary Table 1

Supplementary Table 2

Supplementary Tables 3, 4, 5 and 6
