# Supplementary material for: Risk of hospitalization from drug-drug interactions in the Elderly: real-world evidence in a large administrative database
Source: Aging (Albany NY). 2020 Oct 5;12(19):19711–39. doi: 10.18632/aging.104018 (PMC7732312; doi:10.18632/aging.104018)
Supplement: Supplementary Figures [file aging-12-104018-s001..pdf]

SUPPLEMENTARY FIGURES

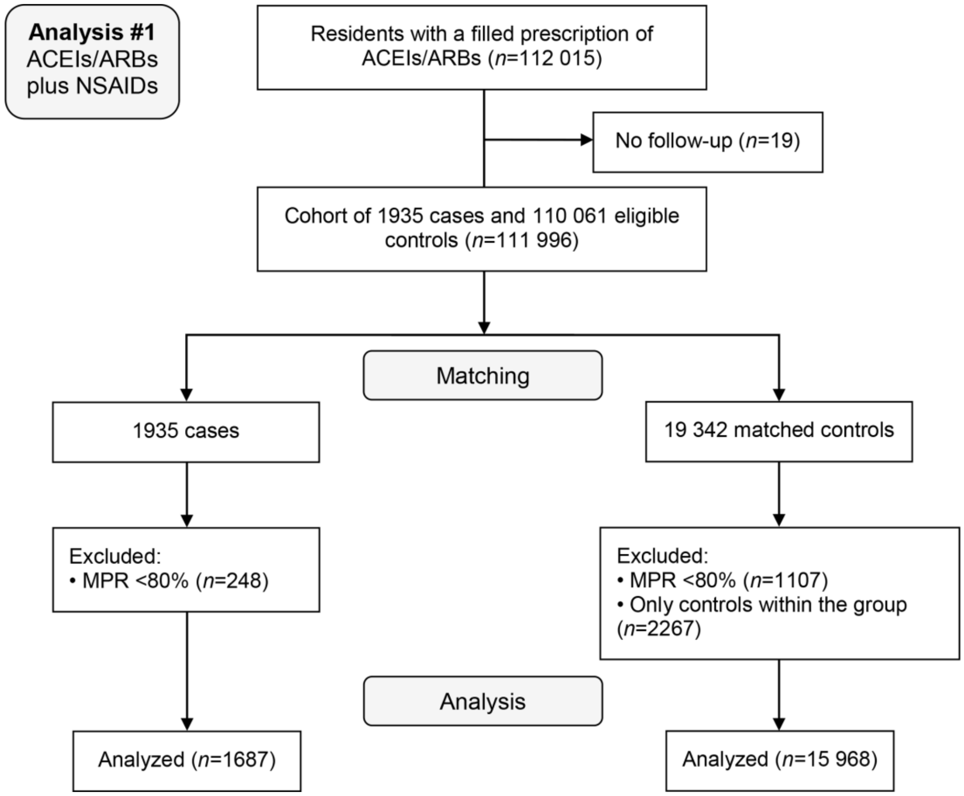

Supplementary Figure 1. Diagram depicting selection of the study population between January 2017 and June 2017.

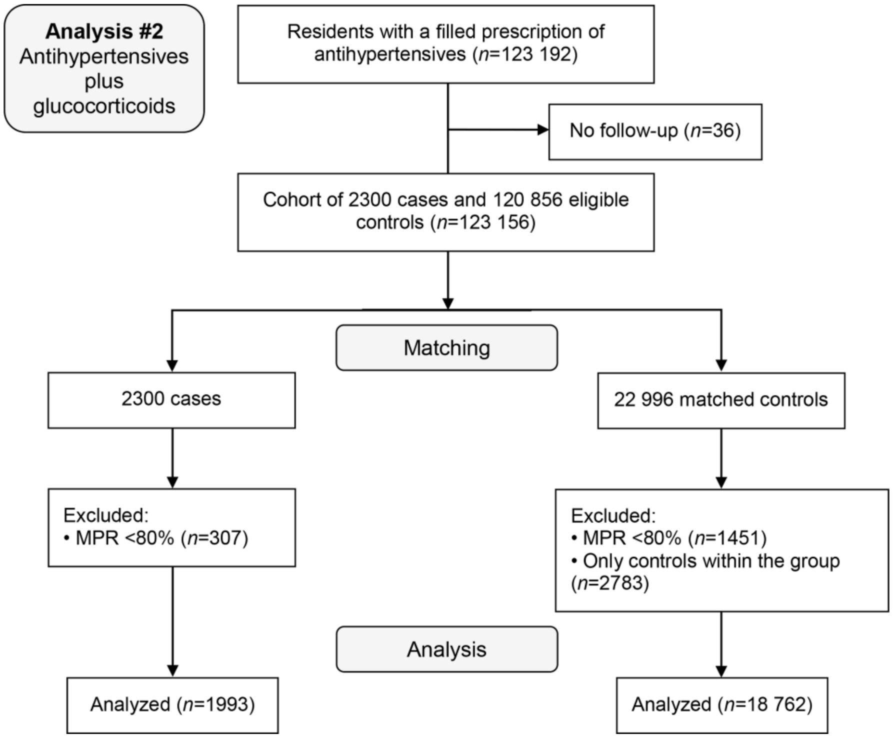

Supplementary Figure 2. Diagram depicting selection of the study population between January 2017 and June 2017.

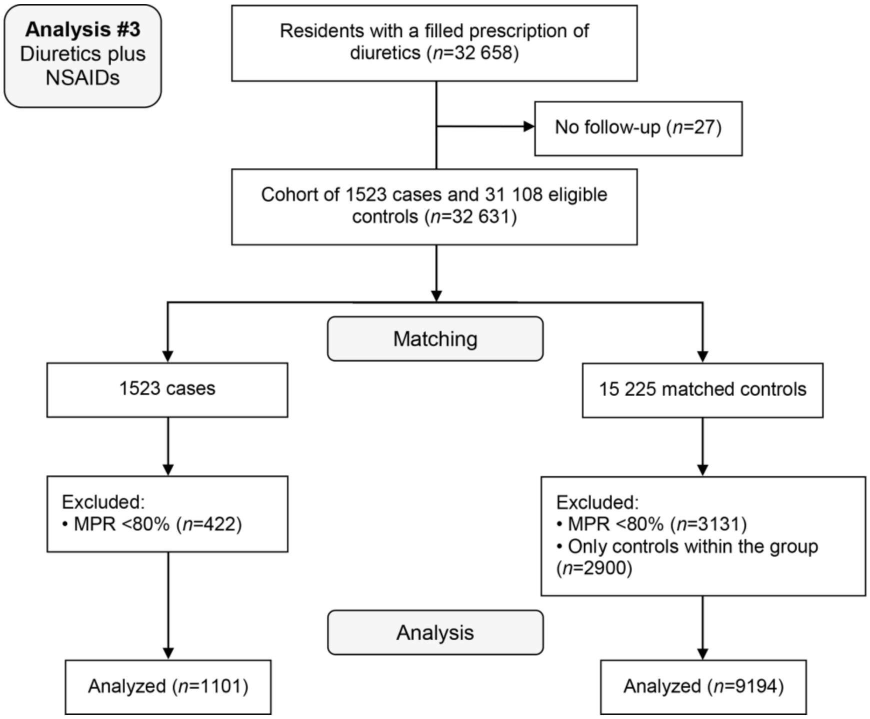

Supplementary Figure 3. Diagram depicting selection of the study population between January 2017 and June 2017.

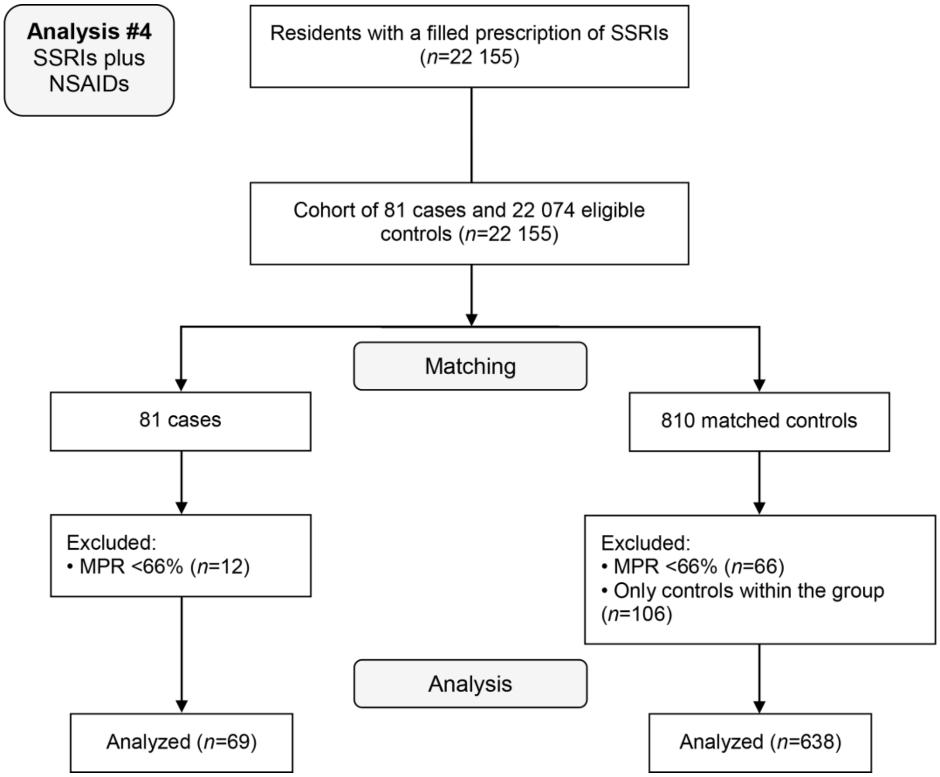

Supplementary Figure 4. Diagram depicting selection of the study population between January 2017 and June 2017.

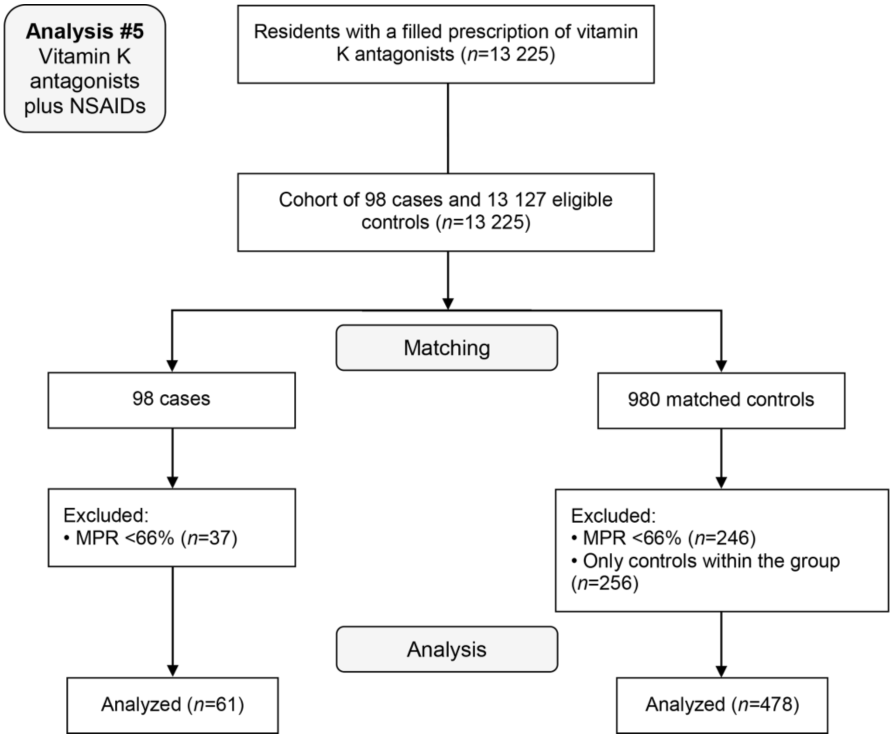

Supplementary Figure 5. Diagram depicting selection of the study population between January 2017 and June 2017.

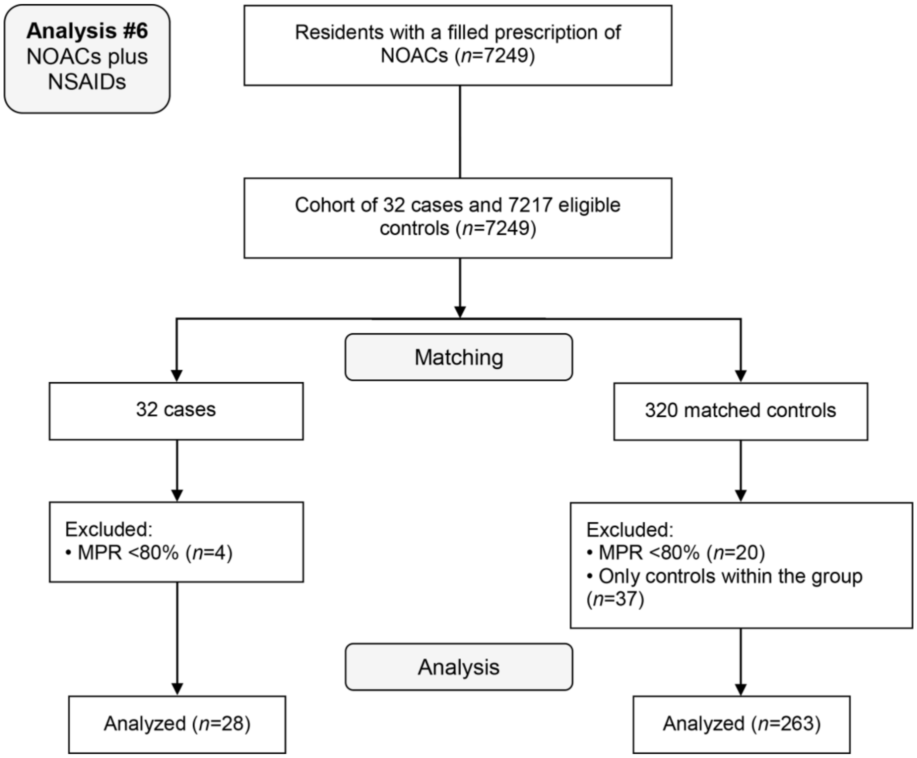

Supplementary Figure 6. Diagram depicting selection of the study population between January 2017 and June 2017.

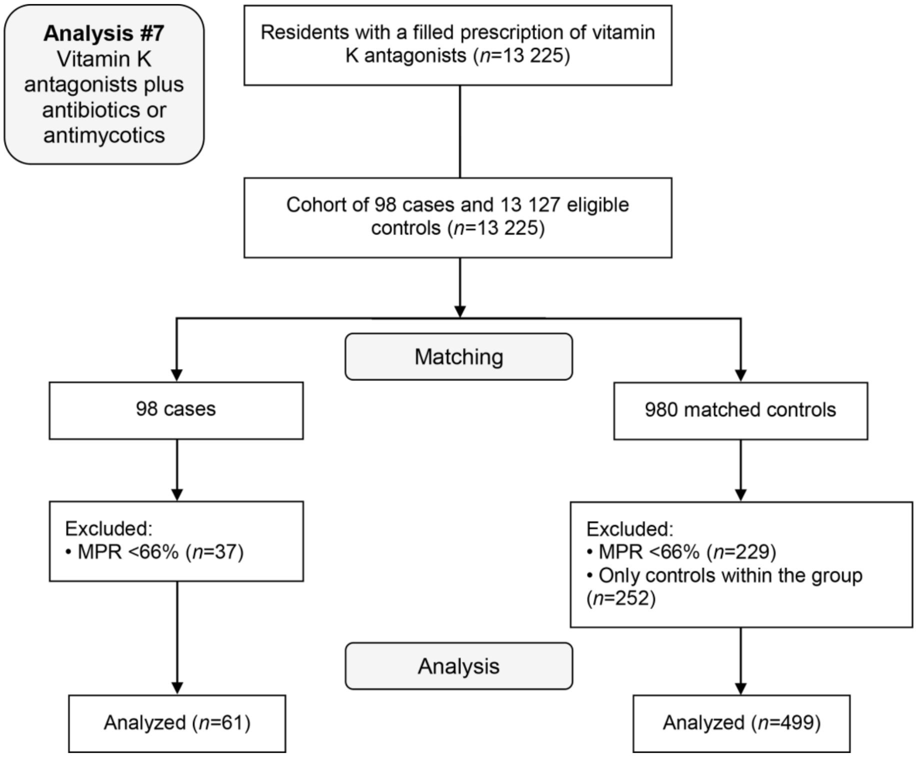

Supplementary Figure 7. Diagram depicting selection of the study population between January 2017 and June 2017.

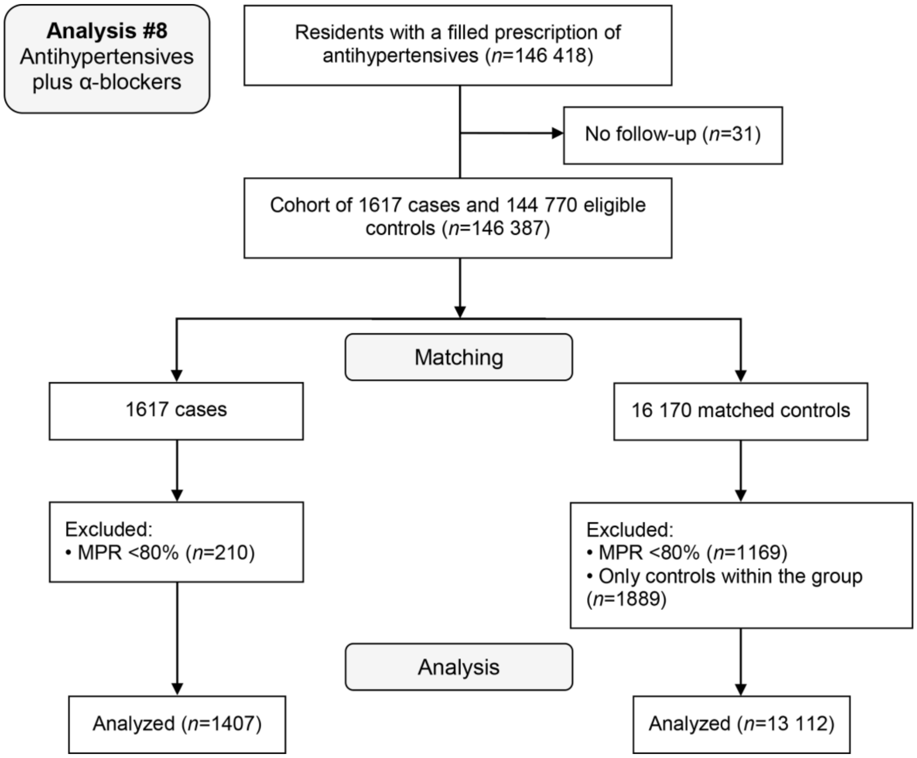

Supplementary Figure 8. Diagram depicting selection of the study population between January 2017 and June 2017.

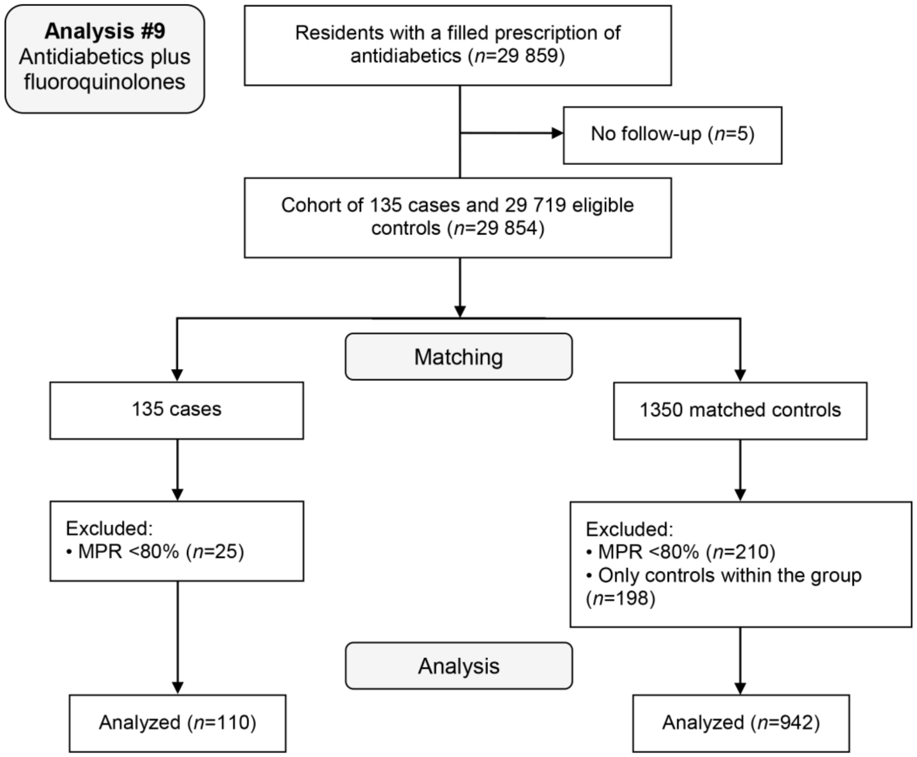

Supplementary Figure 9. Diagram depicting selection of the study population between January 2017 and June 2017.

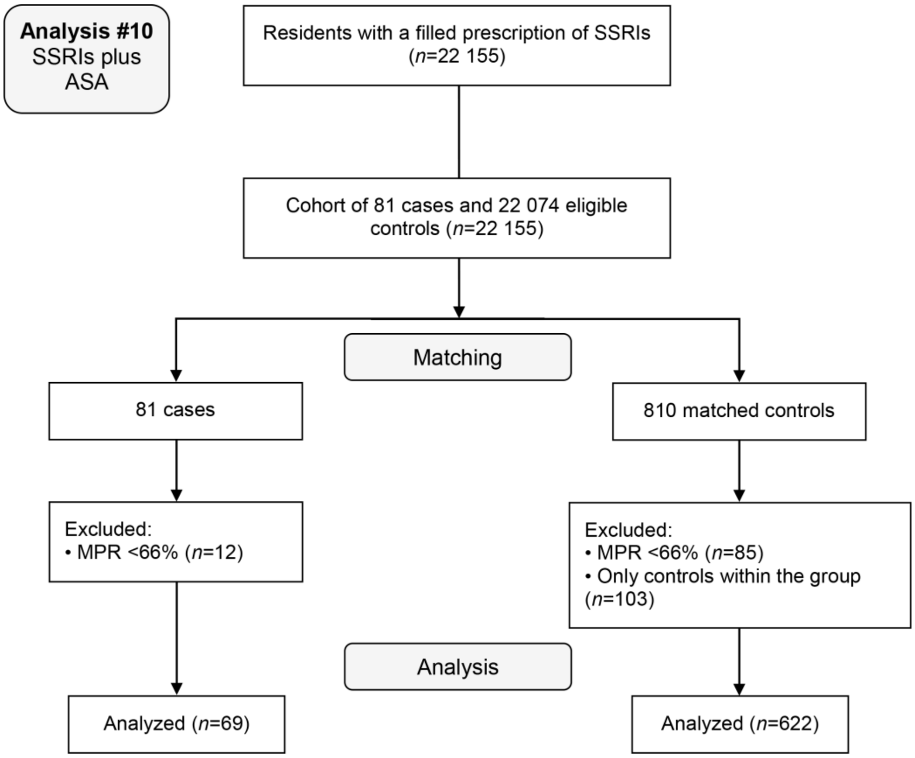

Supplementary Figure 10. Diagram depicting selection of the study population between January 2017 and June 2017.

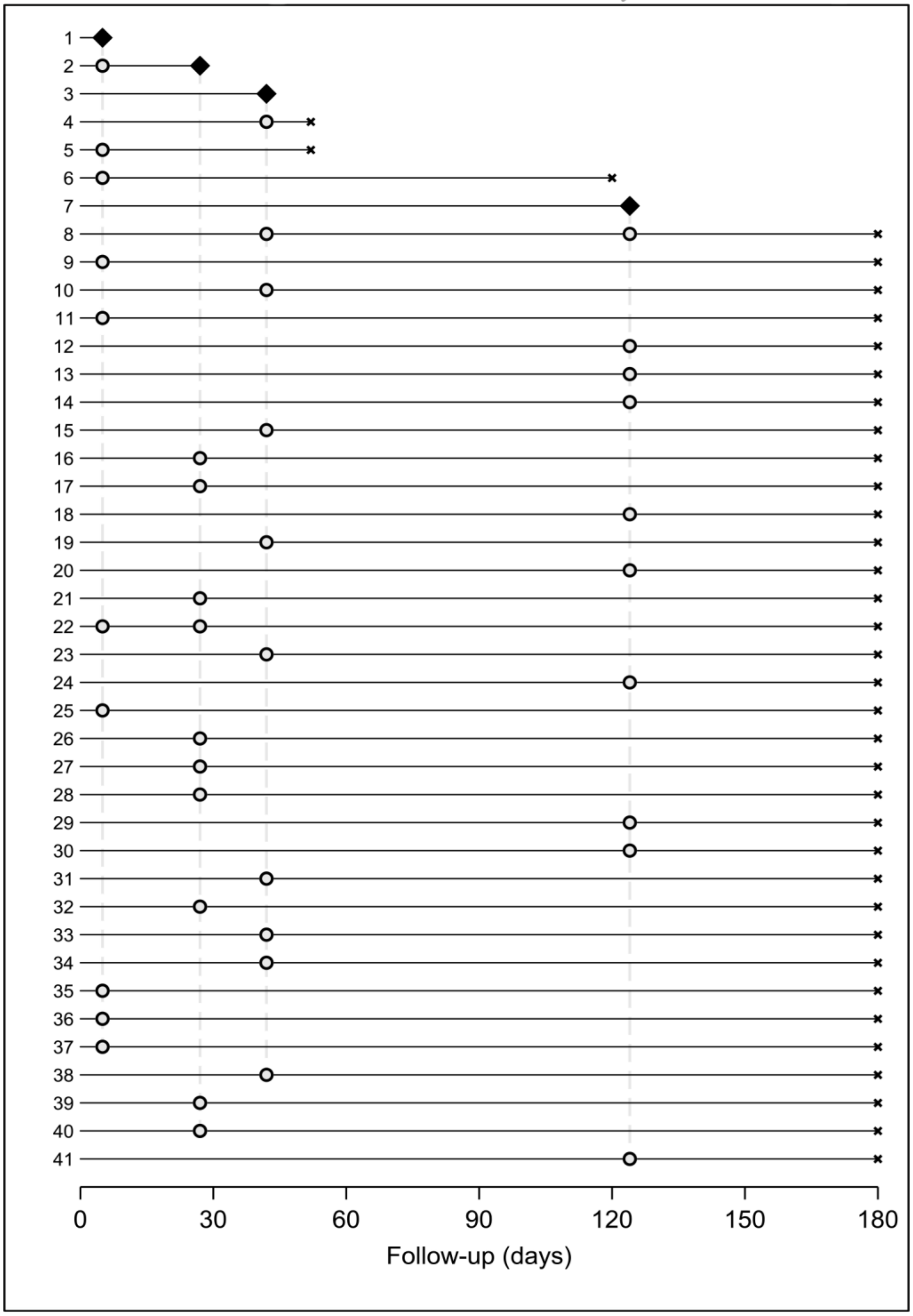

Supplementary Figure 11. An illustrative example of the nested case-control design used in each interaction analysis. There are ten controls (*gray circles*) for every case (*black diamonds*). Follow-up periods for all the members of the cohort (*horizontal black lines*) begin at the zero-time on the time axis, which is the first date of a dispensed prescription of a chronic drug. Deaths (patients #4, #5 and #6) or no events before the end of the follow-up are treated as censored events (*x signs*). Controls are randomly selected from the risk sets (*vertical dashed lines*) of each case. A future case may be selected as a control for a prior case (patient #2), and a subject may be selected as a control for two different cases (patients #8 and #22). Drug-drug interactions are investigated between the cohort entry and the matching date.
