# Supplementary material for: Risk of hospitalization from drug-drug interactions in the Elderly: real-world evidence in a large administrative database
Source: Aging (Albany NY). 2020 Oct 5;12(19):19711–39. doi: 10.18632/aging.104018 (PMC7732312; doi:10.18632/aging.104018)
Supplement: Supplementary Table 1 [file aging-12-104018-s002..docx]

**Supplementary Table 1.** DDI-related hospital admissions during follow-up (study outcomes), by interaction analysis.

| # | Interaction analysis | Follow-up hospitalizations | ICD-9-CM/(Outpatient Care Database) code |
| --- | --- | --- | --- |
| 1 | ACEIs/ARBs plus NSAIDs | Hyponatremia | 276.1 |
|  |  | Hyperkalemia | 276.7 |
|  |  | Hypertensive crisis | 401.xx-405.xx |
|  |  | Acute myocardial infarction | 410.xx |
|  |  | Heart failure | 428.xx |
|  |  | Cerebrovascular event | 430.xx-438.xx |
|  |  | Acute kidney failure | 584.xx |
|  |  | *(Computed tomography of the head)* | *(87.03, 87.03.1)* |
|  |  | *(Nephrology visit)* | *(89.01.B, 89.7B.4)* |
| 2 | ACEIs/ARBs or diuretics plus glucocorticoids | Hyponatremia | 276.1 |
|  |  | Hyperkalemia | 276.7 |
|  |  | Hypertensive crisis | 401.xx-405.xx |
|  |  | Acute myocardial infarction | 410.xx |
|  |  | Heart failure | 428.xx |
|  |  | Cerebrovascular event | 430.xx-438.xx |
|  |  | Acute kidney failure | 584.xx |
|  |  | *(Computed tomography of the head)* | *(87.03, 87.03.1)* |
|  |  | *(Nephrology visit)* | *(89.01.B, 89.7B.4)* |
| 3 | Diuretics plus NSAIDs | Hyponatremia | 276.1 |
|  |  | Hyperkalemia | 276.7 |
|  |  | Hypertensive crisis | 401.xx-405.xx |
|  |  | Acute myocardial infarction | 410.xx |
|  |  | Heart failure | 428.xx |
|  |  | Cerebrovascular event | 430.xx-438.xx |
|  |  | Acute kidney failure | 584.xx |
|  |  | *(Computed tomography of the head)* | *(87.03, 87.03.1)* |
|  |  | *(Nephrology visit)* | *(89.01.B, 89.7B.4)* |
| 4 | SSRIs plus NSAIDs | Intracranial bleeding | 430.xx, 431.xx, 432.xx |
|  |  | Gastrointestinal bleeding | 578.xx, 569.3x, 562.12, 535.71, 535.61, 535.51, 535.41, 535.31, 535.21, 535.11, 535.01, 534.6x, 534.4x, 534.2x, 534.0x, 533.6x, 533.4x, 533.4x, 533.2x, 533.0x, 532.6x, 532.4x, 532.2x, 532.0x, 531.6x, 531.4x, 531.2x, 531.0x, 530.82, 530.7x, 530.4x, 530.21, 459.0x |
|  |  | Other hemorrhagic diathesis | 287.9x, 287.8x, 286.5x |
|  |  | *(Gastroenterology visit)* | *(89.01.9, 89.7A.9)* |
|  |  | *(Esophagogastroduodenoscopy)* | *(42.24, 44.14, 45.13, 45.14, 45.16)* |
|  |  | *(Colonoscopy)* | *(45.23, 45.24, 45.25)* |
|  |  | *(Rectosigmoidoscopy)* | *(45.24, 45.25, 48.23, 48.24)* |
|  |  | *(Computed tomography of the head)* | *(87.03, 87.03.1)* |
| 5 | Vitamin K antagonists plus NSAIDs | Intracranial bleeding | 430.xx, 431.xx, 432.xx |
|  |  | Gastrointestinal bleeding | 578.xx, 569.3x, 562.12, 535.71, 535.61, 535.51, 535.41, 535.31, 535.21, 535.11, 535.01, 534.6x, 534.4x, 534.2x, 534.0x, 533.6x, 533.4x, 533.4x, 533.2x, 533.0x, 532.6x, 532.4x, 532.2x, 532.0x, 531.6x, 531.4x, 531.2x, 531.0x, 530.82, 530.7x, 530.4x, 530.21, 459.0x |
|  |  | Other hemorrhagic diathesis | 287.9x, 287.8x, 286.5x |
|  |  | *(Gastroenterology visit)* | *(89.01.9, 89.7A.9)* |
|  |  | *(Esophagogastroduodenoscopy)* | *(42.24, 44.14, 45.13, 45.14, 45.16)* |
|  |  | *(Colonoscopy)* | *(45.23, 45.24, 45.25)* |
|  |  | *(Rectosigmoidoscopy)* | *(45.24, 45.25, 48.23, 48.24)* |
|  |  | *(Computed tomography of the head)* | *(87.03, 87.03.1)* |
| 6 | NOACs plus NSAIDs | Intracranial bleeding | 430.xx, 431.xx, 432.xx |
|  |  | Gastrointestinal bleeding | 578.xx, 569.3x, 562.12, 535.71, 535.61, 535.51, 535.41, 535.31, 535.21, 535.11, 535.01, 534.6x, 534.4x, 534.2x, 534.0x, 533.6x, 533.4x, 533.4x, 533.2x, 533.0x, 532.6x, 532.4x, 532.2x, 532.0x, 531.6x, 531.4x, 531.2x, 531.0x, 530.82, 530.7x, 530.4x, 530.21, 459.0x |
|  |  | Other hemorrhagic diathesis | 287.9x, 287.8x, 286.5x |
|  |  | *(Gastroenterology visit)* | *(89.01.9, 89.7A.9)* |
|  |  | *(Esophagogastroduodenoscopy)* | *(42.24, 44.14, 45.13, 45.14, 45.16)* |
|  |  | *(Colonoscopy)* | *(45.23, 45.24, 45.25)* |
|  |  | *(Rectosigmoidoscopy)* | *(45.24, 45.25, 48.23, 48.24)* |
|  |  | *(Computed tomography of the head)* | *(87.03, 87.03.1)* |
| 7 | Vitamin K antagonists plus antibiotics or antimycotics | Intracranial bleeding | 430.xx, 431.xx, 432.xx |
|  |  | Gastrointestinal bleeding | 578.xx, 569.3x, 562.12, 535.71, 535.61, 535.51, 535.41, 535.31, 535.21, 535.11, 535.01, 534.6x, 534.4x, 534.2x, 534.0x, 533.6x, 533.4x, 533.4x, 533.2x, 533.0x, 532.6x, 532.4x, 532.2x, 532.0x, 531.6x, 531.4x, 531.2x, 531.0x, 530.82, 530.7x, 530.4x, 530.21, 459.0x |
|  |  | Other hemorrhagic diathesis | 287.9x, 287.8x, 286.5x |
|  |  | *(Gastroenterology visit)* | *(89.01.9, 89.7A.9)* |
|  |  | *(Esophagogastroduodenoscopy)* | *(42.24, 44.14, 45.13, 45.14, 45.16)* |
|  |  | *(Colonoscopy)* | *(45.23, 45.24, 45.25)* |
|  |  | *(Rectosigmoidoscopy)* | *(45.24, 45.25, 48.23, 48.24)* |
|  |  | *(Computed tomography of the head)* | *(87.03, 87.03.1)* |
| 8 | Antihypertensives plus α-blockers | Syncope | 780.2, 992.1 |
|  |  | Fracture | 800-829 |
|  |  | Dislocation | 830-839 |
|  |  | Sprain | 840-848 |
|  |  | Intracranial trauma | 850-854 |
|  |  | Intra-abdominal trauma | 860-869 |
|  |  | After-effects of trauma contusion | 905-908 |
|  |  | Contusion | 920-924 |
|  |  | Orthostatic hypotension | 458.0, 458.29, 458.8, 458.9, 785.50-785.59, 796.3 |
| 9 | Antidiabetics plus fluoroquinolones | Complication of secondary diabetes | 249.xx |
|  |  | Complications of diabetes | 250.xx |
|  |  | Hypoglycemic coma | 251.0, 251.2 |
|  |  | Glycemic abnormalities | 790.2x |
|  |  | Dietary surveillance and counseling | V65.3 |
|  |  | *(Diabetes visit)* | *(890180, 897A80)* |
| 10 | SSRIs plus ASA | Intracranial bleeding | 430.xx, 431.xx, 432.xx |
|  |  | Gastrointestinal bleeding | 578.xx, 569.3x, 562.12, 535.71, 535.61, 535.51, 535.41, 535.31, 535.21, 535.11, 535.01, 534.6x, 534.4x, 534.2x, 534.0x, 533.6x, 533.4x, 533.4x, 533.2x, 533.0x, 532.6x, 532.4x, 532.2x, 532.0x, 531.6x, 531.4x, 531.2x, 531.0x, 530.82, 530.7x, 530.4x, 530.21, 459.0x |
|  |  | Other hemorrhagic diathesis | 287.9x, 287.8x, 286.5x |
|  |  | *(Gastroenterology visit)* | *(89.01.9, 89.7A.9)* |
|  |  | *(Esophagogastroduodenoscopy)* | *(42.24, 44.14, 45.13, 45.14, 45.16)* |
|  |  | *(Colonoscopy)* | *(45.23, 45.24, 45.25)* |
|  |  | *(Rectosigmoidoscopy)* | *(45.24, 45.25, 48.23, 48.24)* |
|  |  | *(Computed tomography of the head)* | *(87.03, 87.03.1)* |
