# Supplementary material for: Risk of hospitalization from drug-drug interactions in the Elderly: real-world evidence in a large administrative database
Source: Aging (Albany NY). 2020 Oct 5;12(19):19711–39. doi: 10.18632/aging.104018 (PMC7732312; doi:10.18632/aging.104018)
Supplement: Supplementary Table 2 [file aging-12-104018-s003..docx]

**Supplementary Table 2.** Interfering drugs, i.e., drugs that could influence the association between DDIs and outcomes. They have been established using specific topics from UpToDate, supplemented by the FDA Adverse Event Reporting System (FAERS) Public Dashboard. Drugs mentioned in UpToDate or FEARS were evaluated for risk of specific outcome. A minimum risk of at least .1% to 1% or the inclusion of contraindication was maintained. For this selection, we used two independent drug information services: Drugs.com and Farmacotherapeutisch kompas (National Health Care Institute of the Netherlands). Some of the interfering drugs can be proxy for disease, and therefore may influence the results.

| 1. Myocardial infarction  Analyses: #1, #2, #3 | |
| --- | --- |
| Group | Interfering drugs & ATC-codes |
| Antidiabetics | Thiazolidinediones (A10BG) |
| NSAIDs | M01A, (salicylacid and derivates: N02BA) |
| Hormonal steroids | Glucocorticoids (H02AB) mineralocorticoids (H02AA), (androgens: G03B, G03E) |
| Immunosuppressive agents | TNF-a-Blockers (L04AB), Alkylating agents (L01A),  Taxenes (L01CD) Ramucirumab (L01XC21), Rituximab (L01XC02), Tacrolimus (L04AD02)  Other immunosuppressives: lenalindomide (L04AX04) pomalidomide (L04AX06),  Selective: fingolimod (L04AA27) |
| Erythropoietin agents | Erythropoietin (B03XA01), Darbepoetin alfa (B03XA02), methoxy polyethylene glycol-epoetin beta (B03XA03), |
| Sex hormones containing estrogens or androgens | G03AA, G03AB, G03B, G03C, G03E, G03F |
| *FAERS search terms: myocardial infarction, myocardial ischemia, acute myocardial infarction.*  *UpToDate: Overview of the acute management of st-elevation myocardial infarction* | |
| 2. Heart failure  Analyses: #1, #2, #3 | |
| Group | Interfering drugs & ATC-codes |
| Immunosuppressive agents | -TNF-a-Blockers (L04AB)  - HER2-targeted agents: trastuzumab(L01XC03), pertuzumab (L01XC13) Rituximab (L01XC02), bevacizumab (L01XC07)  -calineurin inhibitor: Tacrolimus (L04AD02)  -other immunosuppers: thalidomide (L04AX02), lenalindomide (L04AX04) pomalidomide (L04AX06)  -selective: belatacept (L04AA28) |
| Steroids | Glucocorticoids (H02AB) mineralocorticoids (H02AA), (androgens: G03B, G03E) |
| Antidiabetics | Thiazolidinediones (A10BG) |
| Non steroid anti-inflammatory agents | M01A, (salicylacid and derivates: N02BA) |
| Anti-aritmics | Selective calcium channel blockers (C08D), sotalol (C07AA07), class I and class III (C01BA-C01BD and C01BG) |
| Chemotherapy | Alkylating agents (L01A), anthracyclines (L01DB) Taxanes (L01CD) |
| Pulmonal arterial hypertension therapy (PAH) | Antihypertensives for PAH (C02KX) treprostinil (B01AC21), |
| Tricyclic Antidepressants | Non-selective monoamine reuptake inhibitors (N06AA) |
| PDE-3-inhibitors | Cilostazol (B01AC23) anagrelide (L01XX35) |
| Erythropoietin agents | Erythropoietin (B03XA01), Darbepoetin alfa (B03XA02), methoxy polyethylene glycol-epoetin beta (B03XA03), |
| *FAERS searchterms: cardiac failure congestive, cardiac failure acute, cardiac failure chronic*  *UpToDate: Drugs that should be avoided or used with caution in patients with heart failure* | |
| 3. Cerebrovascular event  Analyses: #1, #2, #3 | |
| Group | Interfering drugs & ATC code |
| Immunosuppressive agents | -Monoclonale antibodies: Bevacizumab (L01XC07) Ramucirumab (L01XC21)  - Calcineurin inhibitor: Tacrolimus (L04AD02) |
| Non steroid anti-inflammatory agents | M01A, (salicylacid and derivates: N02BA) |
| Erythropoietin agents | Erythropoietin (B03XA01), Darbepoetin alfa (B03XA02), methoxy polyethylene glycol-epoetin beta (B03XA03), |
| Sex hormones containing estrogens or androgens | G03AA, G03AB, G03B, G03C, G03E, G03F |
| Hormonal steroids | Glucocorticoids (H02AB) mineralocorticoids (H02AA) |
| *FAERS searchterms: cerebral infarction, cerebellar infarction, brain Stem infarction, ischemic cerebral infarction, lacunar infarction, basal ganglia infarction* | |
| 4. Hyperkalemia (potassium)  Analyses: #1, #2, #3 | |
| Group | Interfering drug and ATC code |
| RAS medication | C09 |
| Diuretics | Aldosterone antagonists (C03DA)  Other potassium-sparing agents (C03DB) |
| NSAIDs | M01A, (salicylacid and derivates: N02BA) |
| Digoxin | C01AA05 |
| Antibiotics | Sulfanomides/trimethroprim (J01EE) |
| Immunosuppressives | Calcineurin inhibitors (L04AD), mycophenolic acid (L04AA06), basiliximab (L04AC02), pomalidomide (L04AX06) |
| Heparins | B01AB |
| *FAERS searchterms: hyperkalemia, blood potassium increased,*  *UpToDate: (1) Ttreatment and prevention of hyperkaiemia in adults (2) Causes and evaluation of hyperkalemia in adults.* | |
| 5. Hyponatriemia (sodium)  Analyses: #1, #2, #3 | |
| Group | Interfering drug and ATC code |
| Diuretics | Low-ceiling diuretics, thiazides (C03AA, C03AH, C03AX), Sulfonamides, plain (C03BA, C03BC, C03BD, C03BK, C03BX) Potassium-sparing agents (C03D, C03E), vasopressin antagonists (C03XA) |
| Antidepressants | SSRIs (N06AB), Non-selective monoamine reuptake inhibitors (N06AA) |
| Anti-epileptics | Carboxamide derivates (N03AF), valproate acid (N03AG01) |
| Antineoplastic agents | -Protein kinase: imatinib (L01XE01), sorafenib (L01XE05), dacomitinib (L01XE47), encorafenib (L01XE46), binimetinib (L01XE41), cobimetrinib (L01XE38), Nintedanib (L01XE31), cabozantinib (L01XE26), trametinib ( L01XE25), ponatimib (L01XE24), dabrafenib (L01XE23), regorafenib ( L01XE21) vandetanib (L01XE12), nilotinib (L01XE08)  -Monoclonale antibodies: bevacizumab (L01XC07), ipilimumab (L01XC11), nivolumab (L01XC17), pembrolizumab (L01XC18), ramucirumab(L01XC21), atezolizumab (L01XC32)  -other: pomalidomide (L04AX06), cisplatin (L01XA01) |
| Antibiotics | Sulfanomides/trimethroprim (J01EE) |
| Vasopressin analogues | H01BA |
| Antipsychotics | quetiapine (N05AH04), Haloperidol (N05AD01), Lurasidone (N05AE05), Pimozide (N05AG02) |
| *FAERS searchterms: hyponatremia, blood sodium decreased*  *UpToDate: (1) Causes of hypotonic hyponatremia in adults (2) Diagnostic evaluation of adults with hyponatremia. (3) pathophysiology and etiology of the syndrome of inappropriate antidiuretic hormone secretion (SIADH)* | |
| 6. Hypertensive (crisis)  Analyses: #1, #2, #3 | |
| Group | Interfering drug and ATC code |
| Immunosuppressive agents | -Calcineurin inhibitors (L04AD)  -TNFalfa blockers (L04AB)  -Monoclonal antibodies (L01XC)  -Selective immunosuppressives (L04AA)  -Tyrosine kinase inhibitor: sunitinib (L01XE04), sorafenib(L01XE05), dasatinib (L01XE06), nilotinib (L01XE08), temsirolimus (L01XE09), everolimus (L01XE10), pazopanib (L01XE11), vandetanib (L01XE12) bosutinib (L01XE14), axitinib (L01XE17), regorafenib (L01XE21), dabrafenib ( L01XE23), pontanib (L01XE24) trametinib L01XE25), cabozantinib (L01XE26) ibrutinib (L01XE27) levantinib (L01XE29), nintedanib (L01XE31), tivozanib (L01XE34) cobimetinib (L01XE38), midostaurin (L01XE39), binimetinib (L01XE41), brigatinib (L01XE43), encorafenib (L01XE46)  -Other immunosupressives: lenalindomide (L04AX04) pomalidomide (L04AX06) |
| Antidepressants  = all antidepressants (N06A) | SSRI (N06AB), Monoamine oxidase B inhibitors (N04BD), Monoamine oxidase B inhibitors (N06AG) Monoamine oxidase inhibitors, non-selective (N06AF) Non-selective monoamine reuptake inhibitors (N06AA) Other antidepressants (N06AX) |
| Sex hormones | G03A, G03B, G03C, G30D, G03E, G03F |
| Erythropoietin | B03XA01 |
| NSAID | M01A, (salicylacid and derivates: N02BA) |
| corticosteroids | Glucocorticoids (H02AB) mineralocorticoids (H02AA) |
| Central acting sympathomimetics | N06BA |
| Antipsychotics | Diazepines, oxazepines, thiazepines and oxepines (N05AH), sulpiride (N05AL01) |
| Adrenergic and dopaminergic agents | C01CA |
| Podophyllotoxin derivatives | Etoposide L01CB01 |
| *FAERS searchterms: hypertension, hypertensive urgency, hypertensive emergency*  *UpToDate: overview of hypertension in adults* | |
| 7. (Acute) kidney failure  Analyses: #1, #2, #3 | |
| Group | Interfering drug and ATC code |
| NSAIDs | M01A, (salicylacid and derivates: N02BA) |
| ACEIs/ARBs | Renin-angiotensin system agents (C09) |
| Antibiotics | Aminoglycoside (J01G) Amphotericin B(J02AA01), Sulfonamides and trimethoprim (J01E, A07AB), glycopeptide antibacterials (J01XA), fluoroquinolones (J01MA), bacitracin (J01XX10), pentamide isethionate (P01CX01) Cefotaxime (J01DD01, J01DD51), cefaclor (J01DC04) |
| Antineoplastic agents | - Anthracyclynes (L01DB)  -platinum agents (L01XA)  - podophyllotoxin derivates (L01CB)  -Alkylating agents: ifosfamide(L01AA06), cyclophosphamide (L01AA01), streptozocin (L01AD04),  -antimetabolites: capecitabine (L01BC06), clofarabine (L01BB06), cytarabine (L01BC01), flurabine cladribine (L01BB04), gemcitabine (L01BC05), hydroxyurea (L01XX05) methotrexate (L04AX03, L01BA01) pemetrexed(L01BA04).  -Taxanes: cabazitaxel (L01CD04)  -Vinca alkaloids: vinflunine (L01CA05)  - eribulin (L01XX41)  -miscellaneous cytotoxic agents: arsenic trioxide (L01XX27) |
| Immunosuppressive agents | -Calcineurin inhibitors (L04AD)  -Monoclonal antibodies: ipilimumab (L01XC11) nivolumab (L01XC17) Pembrolizumab (L01XC18)  -TNFa blockers: adalimumab (L04AB04)  -Other: lenalindomide (L04AX04) pomalidomide (L04AX06)  -Selective immunosuppressives: eculizumab (L04AA25)  -Protease inhibitors: carfilzomib (L01XX45) |
| Nucleoside and nucleoside reverse transcriptase inhibitors | J05AF |
| Antidiabetics | Metformin (A10BA02, A10BD02, A10BD03, A10BD05, A10BD07, A10BD08, A10BD10, A10BD11, A10BD13, A10BD14, A10BD15, A10BD16, A10BD17, A10BD18, A10BD20, A10BD22, A10BD23, A10BD25), Canafliflozin (A10BK02) |
| Lithium | N05AN |
| Other antimycotics for systemic use | J02AX |
| Non-selective β-blocking agents | C07AG, C07AA |
| Direct factor Xa inhibitor | Rivaroxaban (B01AF01) |
| Gold preparations | M01CB |
| *FAERS searchterms: acute kidney injury, renal failure, renal injury, prerenal failure, postrenal failure*  *UpToDate: (1) Overview of the management of acute kidney injury (AKI) in adults. (2) Overview of the management of chronic kidney disease in adults. (3) Chemotherapy nephrotoxicity and dose modification in patients with renal insufficiency: conventional cytotoxic agents.* | |
| 8. Gastrointestinal bleeding  Analyses: #4, #5, #6, #7, #10 | |
| Group | Interfering drugs and ATC code |
| NSAIDs | M01A (salicylacid and derivates: N02BA) |
| Antidepressants | SSRIs (N06AB) |
| Corticosteroids | Glucocorticoids (H02AB) mineralocorticoids (H02AA) |
| Immunosuppressives | -Antracyclinederivates (L01DB)-Monoclonal antibodies: bevacizumab (L01XC07) Gemtuzumab ozogamicin (L01XC05), ipilimumab (L01XC11)  Ramucirumab (L01XC21) Inotuzumab ozogamicine (L01XC26)  -TNFa blockers: infliximab (L04AB02)  -Calcineurin inhibitors: Tacrolimus (L04AD02)  -Other immunosuppressants: methotrexaat (L04AX03, L01BA01), lenalindomide (L04AX04) pomalidomide (L04AX06)  -Selective: belatacept (L04AA28) |
| Antithrombotic agents | B01A |
| Pyrimidine analogues | L01BC |
| *FAERS searchterms: upper gastrointestinal hemorrhage, lower gastrointestinal hemorrhage, ulcer hemorrhage, gastrointestinal ulcer*  *UpToDate: (1) Causes of upper gastrointestinal bleeding in adults. (2) Approach to acute gastrointestinal bleeding in adults.* | |
| 9. Intracranial bleeding  Analyses: #4, #5, #6, #7, #10 | |
| Group | Interfering drug and ATC code |
| Antithrombotic agents | B01A |
| Immunosuppressants | Monoclonal antibodies Inotuzumab ozogamicine (L01XC26)  Calcineurin inhibitor: Tacrolimus (L04AD02)  Other immunosuprress: pomalidomide (L04AX06) |
| Pyrimidine analogues | L01BC |
| Podophyllotoxin derivatives | Etoposide (L01CB01) |
| Anthracyclines | L01DB |
| *FAERS searchterms: hemorrhage intracranial, hemorrhagic stroke, basal ganglia hemorrhage, brainstem hemorrhage, cerebellar hemorrhage, cerebral hemorrhage.*  *UpToDate: Spontaneous intracerebral hemorrhage: pathogenesis, clinical features, and diagnosis* | |
| 10. Bleeding  Analyses: #4, #5, #6, #7, #10 | |
| Group | Interfering drug and ATC code |
| Antithrombotic agents | B01A |
| Glucocorticoids | H02AB, (inhalatie: R03BA) |
| SSRIs | N06AB |
| Immunosuppressant | -Monoclonal antibodies: Rituximab (L01XC02), Trastuzumab (L01XC03), Gemtuzumab ozogamicin (L01XC05), Bevacizumab (L01XC05), panitumumab (L01XC08), [pertuzumab](https://www.whocc.no/atc_ddd_index/?code=L01XC13&showdescription=yes) (L01XC13) [trastuzumab emtansine](https://www.whocc.no/atc_ddd_index/?code=L01XC14&showdescription=yes)  (L01XC14), Ramucirumab (L01XC21), Inotuzumab ozogamicine (L01XC26 )  -TNFa: [certolizumab pegol](https://www.whocc.no/atc_ddd_index/?code=L04AB05&showdescription=yes) L04AB05  -Calcineurin inhibitor: Tacrolimus (L04AD02)  -Other immunosuppressants: lenalindomide (L04AX04) pomalidomide (L04AX06)  -Selectieve immunosuppressives: sirolimus (L04AA10) eculizumab (L04AA25) alemtuzumab (L04AA34) |
| Pyrimidine analogues | L01BC |
| Podophyllotoxin derivatives | Etoposide (L01CB01) |
| Sex hormones | G03A, G03C, G03D, G03F, G03E |
| *FAERS searchterms: hemorrhage*  *UpToDate: Approach to the adult with a suspected bleeding disorder* | |
| 11. (Orthostatic)hypotension/syncope  Analysis: #8 | |
| Group | Interfering drug and ATC code |
| Antihypertensives | Betablocking agents (C07A), Renin-angiotensin system agents (C09), diuretics (C03), peripheral vasodilators (C04), betablocking agents (C07), calciumchannel blockers (C08) |
| Antidepressants | N06A |
| Opioids | N01AH, N02A |
| Antipsychotics | N05A |
| chemotherapy | Etoposide L01CB01 |
| Anti-Parkinson drugs | Dopaminergic agents (N04B) |
| Immunosuppressant agents | Monoclonal antibodies: hypotensie in algemeen!  Calcineurin: Tacrolimus (L04AD02)  Other: lenalindomide L04AX04) pomalidomide (L04AX06)  Selectieve immunosuppressive: abatacept (L04AA24), eculizumab (L04AA25), belimumab (L04AA26), belatacept (L04AA28), ocrelizumab (L04AA36) |
| Vasodilators | C01D |
| Antiarrhythmics class I and III | C01B |
| *FAERS searchterms: orthostatic hypotension, blood pressure orthostatic, syncope, fall,*  *UpToDate: syncope in adults: epidemiology, pathogenesis, and etiologies.* | |
| 12. Glucose changes (hypo/hyperglycemia) and diabetic coma  Analysis: #9 | |
| Group | Interfering drug and ATC code |
| Anti-infectives | Protease ihibitors (J05AE), NRTIs (J05AF), Pentamidine (P01CX01), |
| Antipsychotics | Olanzapine (N05AH03) paliperidone (N05AX13) quetiapine (N05AH04), risperidone (N05AX08) aripiprazole (N05AX12) cariprazine (N05AX15) brexpiprazole (N05AX16) iloperidone(N05AX14) amisulpride (N05AL05) lithium (N05AN01) |
| Cardiovascular | Non-selective betablocking agents (C07AA), statins (C10AA, C10B),  Thiazide diuretics (C03AA), chlortalidone (C03BA04), indapamide (C03BA11), diazoxide (V03AH01), |
| Gonadotropin releasing hormone analogues | L02AE |
| Glucocorticoids | H02AB |
| Sex hormones | Progrestogens and estrogens (G03A, G03C, G03D, G03F) |
| Immunosuppressors | Calcineurin inhibitors (L04AD) sirolimus (L04AA10) everolimus (L04AA18) belatacept (L04AA28) |
| Somatropine-agonists | H01AC |
| *FAERS searchterms: hypoglycemia, hyperglycemia, hypoglycemic unconsciousness, hypoglycemic coma, hyperglycemic unconsciousness, diabetic hyperglycemic coma, diabetic ketoacidotic hyperglycemic coma*  *UpToDate: (1) Pathogenesis of type 2 diabetes mellitus. (2) Drugs other ten antihyperglycemic agents and alcohol reported to cause hypoglycemia* | |
