# Supplementary material for: Risk of hospitalization from drug-drug interactions in the Elderly: real-world evidence in a large administrative database
Source: Aging (Albany NY). 2020 Oct 5;12(19):19711–39. doi: 10.18632/aging.104018 (PMC7732312; doi:10.18632/aging.104018)
Supplement: Supplementary Tables 3, 4, 5 and 6 [file aging-12-104018-s004..pdf]

SUPPLEMENTARY TABLES

Supplementary Table 3. Hospital admissions for high-risk conditions 3-years prior to cohort entry, by interaction analysis.

#	Interaction analysis	Previous hospitalizations	ICD-9-CM code
1	ACEIs/ARBs plus NSAIDs	Hypertensive crisis	401.xx-405.xx
		Acute myocardial infarction	410.xx
		Heart failure	428.xx
		Kidney failure	580.xx-589.xx
2	ACEIs/ARBs or diuretics plus glucocorticoids	Hypertensive crisis	401.xx-405.xx
		Acute myocardial infarction	410.xx
		Heart failure	428.xx
		Kidney failure	580.xx-589.xx
3	Diuretics plus NSAIDs	Hypertensive crisis	401.xx-405.xx
		Acute myocardial infarction	410.xx
		Heart failure	428.xx
		Kidney failure	580.xx-589.xx
4	SSRIs plus NSAIDs	Hypertensive crisis	401.xx-405.xx
		Cerebrovascular event	430.xx-438.xx
		Intracranial bleeding	430.xx, 431.xx, 432.xx
		Gastrointestinal bleeding	578.xx, 569.3x, 562.12, 535.71, 535.61, 535.51, 535.41, 535.31, 535.21, 535.11, 535.01, 534.6x, 534.4x, 534.2x, 534.0x, 533.6x, 533.4x, 533.4x, 533.2x, 533.0x, 532.6x, 532.4x, 532.2x, 532.0x, 531.6x, 531.4x, 531.2x, 531.0x, 530.82, 530.7x, 530.4x, 530.21, 459.0x
		Other hemorrhagic diathesis	287.9x, 287.8x, 286.5x
5	Vitamin K antagonists plus NSAIDs	Hypertensive crisis	401.xx-405.xx
		Cerebrovascular event	430.xx-438.xx
		Intracranial bleeding	430.xx, 431.xx, 432.xx
		Gastrointestinal bleeding	578.xx, 569.3x, 562.12, 535.71, 535.61, 535.51, 535.41, 535.31, 535.21, 535.11, 535.01, 534.6x, 534.4x, 534.2x, 534.0x, 533.6x, 533.4x, 533.4x, 533.2x, 533.0x, 532.6x, 532.4x, 532.2x, 532.0x, 531.6x, 531.4x, 531.2x, 531.0x, 530.82, 530.7x, 530.4x, 530.21, 459.0x
		Other hemorrhagic diathesis	287.9x, 287.8x, 286.5x
6	NOACs plus NSAIDs	Hypertensive crisis	401.xx-405.xx
		Cerebrovascular event	430.xx-438.xx
		Intracranial bleeding	430.xx, 431.xx, 432.xx
		Gastrointestinal bleeding	578.xx, 569.3x, 562.12, 535.71, 535.61, 535.51, 535.41, 535.31, 535.21, 535.11, 535.01, 534.6x, 534.4x, 534.2x, 534.0x, 533.6x, 533.4x, 533.4x, 533.2x, 533.0x, 532.6x, 532.4x, 532.2x, 532.0x,

			531.6x, 531.4x, 531.2x, 531.0x, 530.82, 530.7x, 530.4x, 530.21, 459.0x
		Other hemorrhagic diathesis	287.9x, 287.8x, 286.5x
7	Vitamin K antagonists plus antibiotics or antimycotics	Hypertensive crisis	401.xx-405.xx
		Cerebrovascular event	430.xx-438.xx
		Intracranial bleeding	430.xx, 431.xx, 432.xx
		Gastrointestinal bleeding	578.xx, 569.3x, 562.12, 535.71, 535.61, 535.51, 535.41, 535.31, 535.21, 535.11, 535.01, 534.6x, 534.4x, 534.2x, 534.0x, 533.6x, 533.4x, 533.4x, 533.2x, 533.0x, 532.6x, 532.4x, 532.2x, 532.0x, 531.6x, 531.4x, 531.2x, 531.0x, 530.82, 530.7x, 530.4x, 530.21, 459.0x
		Other hemorrhagic diathesis	287.9x, 287.8x, 286.5x
8	Antihypertensives plus α-blockers	Syncope	780.2, 992.1
		Orthostatic hypotension	458.0, 458.29, 458.8, 458.9, 785.50-785.59, 796.3
9	Antidiabetics plus fluoroquinolones	-	-
10	SSRIs plus ASA	Hypertensive crisis	401.xx-405.xx
		Cerebrovascular event	430.xx-438.xx
		Intracranial bleeding	430.xx, 431.xx, 432.xx
		Gastrointestinal bleeding	578.xx, 569.3x, 562.12, 535.71, 535.61, 535.51, 535.41, 535.31, 535.21, 535.11, 535.01, 534.6x, 534.4x, 534.2x, 534.0x, 533.6x, 533.4x, 533.4x, 533.2x, 533.0x, 532.6x, 532.4x, 532.2x, 532.0x, 531.6x, 531.4x, 531.2x, 531.0x, 530.82, 530.7x, 530.4x, 530.21, 459.0x
		Other hemorrhagic diathesis	287.9x, 287.8x, 286.5x

Supplementary Table 4. Odds ratios (ORs) of hospitalization associated with current (last month) and past (≥2 months before) exposure to DDI.

#	Interaction analysis	Exposure to DDI	Cases	Matched controls	Adjusted OR (95% CI)	Users	
						Prevalent	Incident
1	ACEIs/ARBs plus NSAIDs	No	1549 (91.8)	14 676 (91.9)	Ref.		
		Past	78 (4.6)	776 (4.9)	0.93 (0.72-1.18)	16 478 (93.3)	1177 (6.7)
		Current	60 (3.6)	516 (3.2)	1.05 (0.80-1.39)		
2	ACEIs/ARBs or diuretics plus glucocorticoids	No	1698 (85.2)	17 299 (92.2)	Ref.		
		Past	142 (7.1)	904 (4.8)	1.36* (1.12-1.64)	19 464 (93.8)	1291 (6.2)
		Current	153 (7.7)	559 (3.0)	2.35* (1.93-2.86)		
3	Diuretics plus NSAIDs	No	1038 (94.3)	8734 (95.0)	Ref.		
		Past	36 (3.3)	228 (2.5)	1.26 (0.87-1.83)	8491 (82.5)	1804 (17.5)
		Current	27 (2.5)	232 (2.5)	0.96 (0.64-1.46)		
4	SSRIs plus NSAIDs	No	58 (84.1)	577 (90.4)	Ref.		
		Past	6 (8.7)	42 (6.6)	1.21 (0.47-3.11)	543 (76.8)	164 (23.2)
		Current	5 (7.2)	19 (3.0)	2.88 (0.97-8.59)		
5	Vitamin K antagonists plus NSAIDs	No	57 (93.4)	467 (97.7)	Ref.		
		Past	2 (3.3)	7 (1.5)	1.88 (0.37-9.59)	424 (78.7)	115 (21.3)
		Current	2 (3.3)	4 (0.8)	7.60 (0.98-58.7)		
6	NOACs plus NSAIDs	No	27 (96.4)	255 (97.0)	Ref.		
		Past	0 (0.0)	5 (1.9)	n/a	224 (77.0)	67 (23.0)
		Current	1 (3.6)	3 (1.1)	4.07 (0.38-43.6)		
7	Vitamin K antagonists plus antibiotics or antimycotics	No	57 (93.4)	449 (90.0)	Ref.		
		Past	1 (1.6)	25 (5.0)	0.28 (0.03-2.17)	449 (80.2)	111 (19.8)
		Current	3 (4.9)	25 (5.0)	0.88 (0.25-3.13)		
8	Antihypertensives plus α-blockers	No	1289 (91.6)	11 958 (91.2)	Ref.		
		Past	54 (3.8)	498 (3.8)	1.01 (0.74-1.37)	13 791 (95.0)	728 (5.0)
		Current	64 (4.5)	656 (5.0)	0.89 (0.67-1.18)		
10	SSRIs plus ASA	No	42 (60.9)	400 (64.3)	Ref.		
		Past	15 (21.7)	119 (19.1)	1.38 (0.71-2.68)	552 (79.9)	139 (20.1)
		Current	12 (17.4)	103 (16.6)	1.21 (0.59-2.49)		

These ORs are adjusted for covariates shown in Table 2 and for prevalent user status (≥1 prescriptions for the drug of interest 6-months prior to cohort entry). Values are counts (percentages) unless stated otherwise. Analysis #9 (antidiabetics plus fluoroquinolones) is not presented because previous use of antidiabetics is included in the first model (Table 3).

* Significant at the 0.05 level

Supplementary Table 5. Indications for NSAIDs use (source: HDRs).

Chronic/acute condition	Indication	ICD-9-CM code
Chronic	Osteoarthrosis	715.xx, 718.0x
	Disorders causing back pain	721.0x, 721.1x, 721.2x, 721.3x, 721.4x, 721.9x, 722.0x, 722.1x, 722.2x, 723.1x, 724.1x, 724.2x, 724.3x, 724.5x,
	Rheumatic diseases, poly-arthropathies, chronic arthritis	711.2x, 713.xx, 714.xx, 719.3x, 720.xx, 725.xx 733.6x,
Acute	Osteomyelitis, pathological fracture	730.xx, 733.1x,
	Infectious Arthritis	7.11.0x, 711.1x, 711.3x, 711.4x, 711.5x, 711.6x, 711.7x, 711.8x, 711.9x
	Chrystal arthropathies	712.xx
	Non-chronic arthropathies	716.xx, 717.xx, 818.2x, 718.3x, 718.4x, 719.4x,
	Soft tissue disorders, esenthesopathies, bursopathies, synovitis	719.2x, 726.xs, 727.xx,
	Muscle related pain with NSAID indication	728.0x, 729.1x, 729.3x, 729.5x,
	Cholelithiasis	574.xx

Chronic diseases checked for 3 years prior to cohort entry; acute diseases checked during follow-up or 1 month prior to cohort entry.

Supplementary Table 6. Odds ratios (ORs) of hospitalization associated with current (last month) and past (≥2 months before) exposure to DDI.

#	Interaction analysis	Exposure to DDI	OR (95% CI)		Patients excluded
			Crude	Adjusted*	
1	ACEIs/ARBs plus NSAIDs	No	Ref.	Ref.	17
		Past	0.93 (0.73-1.18)	0.93 (0.73-1.19)	
		Current	1.11 (0.84-1.46)	1.06 (0.80-1.40)	
2	ACEIs/ARBs or diuretics plus glucocorticoids	No	Ref.	Ref.	23
		Past	1.55† (1.29-1.87)	1.36† (1.12-1.65)	
		Current	2.91† (2.41-3.52)	2.38† (1.95-2.90)	
3	Diuretics plus NSAIDs	No	Ref.	Ref.	19
		Past	1.17 (0.81-1.68)	1.27 (0.88-1.84)	
		Current	0.94 (0.63-1.41)	0.97 (0.64-1.46)	
4	SSRIs plus NSAIDs	No	Ref.	Ref.	0
		Past	1.34 (0.53-3.39)	1.21 (0.47-3.08)	
		Current	2.62 (0.95-7.22)	2.88 (0.97-8.59)	
5	Vitamin K antagonists plus NSAIDs	No	Ref.	Ref.	1
		Past	2.14 (0.44-10.4)	2.03 (0.37-11.1)	
		Current	5.52 (0.90-33.8)	7.00 (0.98-59.7)	
6	NOACs plus NSAIDs	No	Ref.	Ref.	2
		Past	n/a	n/a	
		Current	2.99 (0.31-28.8)	3.61 (0.35-37.2)	
7	Vitamin K antagonists plus antibiotics or antimycotics	No	Ref.	Ref.	1
		Past	0.27 (0.04-2.04)	0.27 (0.03-2.09)	
		Current	0.98 (0.29-3.31)	0.82 (0.23-2.89)	
8	Antihypertensives plus α-blockers	No	Ref.	Ref.	12
		Past	0.98 (0.73-1.33)	1.00 (0.74-1.36)	
		Current	0.90 (0.68-1.19)	0.89 (0.67-1.19)	
9	Antidiabetics plus fluoroquinolones	No	Ref.	Ref.	1
		Past	2.00 (0.83-4.77)	2.00 (0.81-4.90)	
		Current	4.54† (1.77-11.7)	4.42† (1.60-11.3)	
10	SSRIs plus ASA	No	Ref.	Ref.	0
		Past	1.17 (0.63-2.19)	1.33 (0.69-2.56)	
		Current	1.12 (0.57-2.21)	1.20 (0.58-2.46)	

These ORs are unbiased estimates of the relative risk of hospitalization. Values are counts (percentages) unless stated otherwise. Patients with >50% of their matched follow-up periods in the hospital are excluded.
* Adjusted for covariates shown in Table 2.
† Significant at the 0.05 level.
